# Enhancing Commercial Antibiotics with Trans-Cinnamaldehyde in Gram-Positive and Gram-Negative Bacteria: An In Vitro Approach

**DOI:** 10.3390/plants13020192

**Published:** 2024-01-10

**Authors:** Natalia Ferrando, María Rosa Pino-Otín, Diego Ballestero, Guillermo Lorca, Eva María Terrado, Elisa Langa

**Affiliations:** 1 Facultad de Ciencias de la Salud, Universidad San Jorge, Campus Universitario Villanueva de Gállego, Autovía A-23 Zaragoza-Huesca, km. 510, 50830 Villanueva de Gállego, Spain; nferrando@usj.es (N.F.); rpino@usj.es (M.R.P.-O.); dballestero@usj.es (D.B.); glorca@usj.es (G.L.); 2 Departamento de Didácticas Específicas, Facultad de Educación, Universisad de Zaragoza, Calle Pedro Cerbuna 12, 50009 Zaragoza, Spain; eterrado@unizar.es

**Keywords:** trans-cinnamaldehyde, antibiotics, synergy, MIC reduction

## Abstract

One strategy to mitigate the emergence of bacterial resistance involves reducing antibiotic doses by combining them with natural products, such as trans-cinnamaldehyde (CIN). The objective of this research was to identify in vitro combinations (CIN + commercial antibiotic (ABX)) that decrease the minimum inhibitory concentration (MIC) of seven antibiotics against 14 different Gram-positive and Gram-negative pathogenic bacteria, most of them classified as ESKAPE. MIC values were measured for all compounds using the broth microdilution method. The effect of the combinations on these microorganisms was analyzed through the checkboard assay to determine the type of activity (synergy, antagonism, or addition). This analysis was complemented with a kinetic study of the synergistic combinations. Fifteen synergistic combinations were characterized for nine of the tested bacteria. CIN demonstrated effectiveness in reducing the MIC of chloramphenicol, streptomycin, amoxicillin, and erythromycin (94–98%) when tested on *Serratia marcescens*, *Staphylococcus aureus*, *Pasteurella aerogenes*, and *Salmonella enterica*, respectively. The kinetic study revealed that when the substances were tested alone at the MIC concentration observed in the synergistic combination, bacterial growth was not inhibited. However, when CIN and the ABX, for which synergy was observed, were tested simultaneously in combination at these same concentrations, the bacterial growth inhibition was complete. This demonstrates the highly potent in vitro synergistic activity of CIN when combined with commercial ABXs. This finding could be particularly beneficial in livestock farming, as this sector witnesses the highest quantities of antimicrobial usage, contributing significantly to antimicrobial resistance issues. Further research focused on this natural compound is thus warranted for this reason.

## 1. Introduction

In the current healthcare environment, the alarming rise in multi-drug-resistant bacterial infections has become a global public health threat. Due to the severity of the issue, the World Health Organization (WHO) declared the spread of antibiotic-resistant bacteria as one of the three greatest public health hazards of the 21st century and published a top-priority pathogen list for which research is of utter importance. Among the highlighted bacteria, *Acinetobacter baumannii*, *Pseudomonas aeruginosa*, *Staphylococcus aureus*, and *Salmonellae* spp. can be found [1].

The spread of antibiotic resistance genes among bacteria [2] has created the necessity of developing alternative therapies or strategies. This approach has led the scientific community to return to the origin of therapies, such as the use of substances naturally synthesized by plants, that is, their primary or secondary metabolites [3]. Plants’ secondary metabolites are involved in vital functions, for instance, growth, development, and storage, and, moreover, they help protect the plant against harmful stress like UV light or different pathogens that can cause infections [4]. Plant secondary metabolites have been (and continue to be) a rich source of bioactive molecules with significant clinical applications. In fact, over 50% of all pharmaceutical drugs currently on the market are directly derived from or inspired by natural products [5]. Some authors, like O’Shea and Moser [6], believed that the historical superiority of novel bioactive natural antimicrobial molecules compared to synthetic libraries was due to evolutionary selection pressure for antibiotic-producing organisms, a higher degree of diversity, and an average increase in heteroatoms. Some examples of unaltered natural compounds that exhibit potent antibacterial activity include penicillin, polymyxin B, or vancomycin [7].

Therefore, the search for new antimicrobial natural products continues to draw the attention of many researchers. One of the most influential families of bioactive phytochemicals are polyphenols, which, alongside terpenes, are one of the most abundant groups of secondary metabolites [8]. The chemical structure of polyphenols includes one hydroxyl group (-OH), responsible not only for the antioxidant properties but also for the antimicrobial ones. OH groups act as proton exchangers, thereby reducing the pH gradient across the plasmatic membrane and thus leading to cell death [9].

For this reason, combinations of conventional antibiotics and natural products with demonstrated antimicrobial properties have been grabbing researchers’ attention [10]. These natural molecules have the potential to increase the antibiotic’s antibacterial effect and, therefore, recover their initial ability to eliminate the most resistant strains of bacteria [11]. The combination effects occur, for instance, by intercalating several simultaneous mechanisms of action, a situation facilitated by the structural and stereochemical complexity of natural active molecules with a wide variety of functional groups [12]. Interaction between two antimicrobial compounds can result in synergy, additive effects, or antagonism. Synergy is generally recognized when the effect of two compounds in combination is stronger than the sum of the effects of each compound when acting alone [13,14]. Such a strategy can also reduce the toxicity of the antibiotic by diminishing its dosage, which can be remarkable in most resistant infections.

Two main reasons to include phytochemicals in pharmacological strategies to solve bacterial resistance problems must be highlighted. Firstly, they are common in nature. The natural environment accounts for a source of very promising ingredients to produce new antimicrobial drugs, in spite of the fact that their antibacterial action is not as effective as that of commercial synthetic drugs [15]. Secondly, most of them had already been used as traditional remedies and, therefore, were presumably safe for humans. In fact, some of them, like cinnamaldehyde, were Generally Recognized As Safe (GRAS) by the Flavoring Extracts Manufacturers’ Association and were approved for food use (21CFR 182.60) by the Food and Drug Administration (FDA), the WHO, and the Council of Europe [16,17,18].

Cinnamaldehyde is a bioactive phytocompound that occurs in the bark of cinnamon trees and is responsible for its flavor and odor. It is a member of the phenylpropanoid family and is produced via the shikimate pathway [19]. Cinnamaldehyde has been deeply studied as a major component of cinnamon due to its numerous properties. Early studies suggest antioxidant properties through inhibition of oxidative stress by elimination of ROS (alkoxyl, superoxide anion, hydroxyl, and peroxyl radicals) responsible for lipid peroxidation, peroxidative hemolysis, and aging of cells, thus preventing oxidative injury that increases cell damage [14,20]. The process of oxidative stress is also linked to coronary and Alzheimer’s disease and some types of cancer [21]. Cinnamaldehyde has also proven to be an efficient anti-inflammatory molecule by inhibiting the intracellular signaling pathway of macrophages, which results in cytokine production suppression [22]. Furthermore, it is also a candidate for anti-diabetic treatments as it has hypoglycemic action thanks to its ability to regulate glucose metabolism [23]. It has an insulinotropic effect due to its ability to alter mRNA expression levels of pyruvate kinase and phosphoenolpyruvate carboxykinase [24]. Moeover, the most relevant property for our study is its antimicrobial activity. Cinnamaldehyde is, in fact, a well-known antimicrobial agent against both Gram-positive and Gram-negative bacteria [25,26].

Despite these facts, few authors have conducted a detailed analysis of the potential synergistic benefits of cinnamaldehyde combined with commercial antimicrobials [11,27]. Thus, the main objective of this research was to identify cinnamaldehyde synergistic combinations with commercial antimicrobials against pathogenic bacteria and to characterize the kinetics of these interactions over time. For this purpose, a group of seven widely used antibiotics with different mechanisms of action and 14 Gram-positive and Gram-negative bacteria responsible for very prevalent infectious pathologies have been selected.

## 2. Results

### 2.1. Toxicity Study

**Figure 1:**
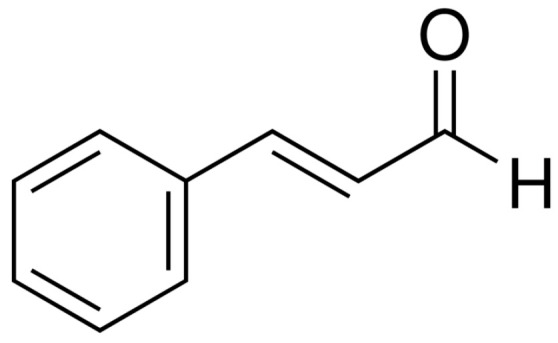
Chemical structure of CIN.

The monoterpene trans-cinnamaldehyde (CIN) (Figure 1) is insoluble in water, and therefore, an organic solvent was needed. To guarantee that the presence of DMSO did not affect bacteria growth, a DMSO toxicity study was developed for each strain as previously described. As it can be seen in Figure S1a,b, DMSO concentrations at or below 2.5% (concentration in the well) can be used without modifying the growth of microorganisms, except for *Proteus mirabilis*, whose survival was 92.7 ± 0.9% at 2.5% of DMSO. For this reason, *P. mirabilis* was excluded in any test where CIN was assayed.

### 2.2. Antimicrobial Susceptibility Test

The MIC results from tests of the natural antimicrobial and conventional antibiotics against the 14 bacterial strains are presented in Table 1. Experiments revealed that the lowest trans-cinnamaldehyde MICs were 500 μg/mL for most strains except for *Enterococcus faecalis* and *Pseudomonas aeruginosa*, whose MICs doubled the previous ones. *P. mirabilis* cannot be probed against this compound because the DMSO concentration required to dissolve it would have affected its growth (Figure S1a).

With these results, some exclusion criteria for the interaction experiments were established as follows:

1. Antibiotics with a MIC for a given bacteria below 10 μg/mL were discarded. These results were considered difficult to improve in combination with a synergistic compound.
2. Bacteria that were susceptible to DMSO at a concentration below 2.5% in the toxicity study were excluded because of the impossibility of solubilizing CIN in pure water.

After applying the mentioned criteria, 60 natural compound-antibiotic–bacteria interactions were selected for the checkboard test.

### 2.3. Checkerboard Assay

The potential reduction of the commercial antibiotic MICs by the natural antimicrobial CIN was examined together with the calculation of the SFIC of the resulting combinations, that is, when both compounds were added simultaneously to the bacterial population. Data on these interactions are offered in Table 2.

Synergistic interactions between the commercial antibiotics and CIN were found for all bacteria tested, except for *A. baumannii*, *E. coli*, *K. aerogenes,* and *P. aeruginosa*, whose interactions were additive and/or antagonistic. Regarding the antibiotics, streptomycin sulfate (STM) was indeed the compound with a higher number of synergistic interactions with CIN (five synergies on *S. enterica, S. aureus, S. agalactiae, L. monocytogenes,* and *E. faecalis*). However, amoxicillin (AMO) and erythromycin (ERY) only displayed this effect on *P. aerogenes* and *S. marcescens*, respectively. On the other hand, the highest antibiotic MIC reduction was found for the antibiotic chloramphenicol (CHL) in the combinations *S. marcescens*-CIN-CHL, with an antibiotic MIC reduction of 98%, and *S. aureus*-CIN-STM, *P. aerogenes*-CIN-AMO, and *S. enterica*-CIN-ERY, where the MIC was reduced by 94%.

The number of synergistic interactions (CIN + ABX) was 15. All of them meant antibiotic MIC reductions higher than 75%. However, some of the interactions coined as ‘additive´ also reported MIC reductions (ranging from 50 to 94%). Hence, if we consider this fact, the number of interactions giving antibiotic MIC reductions grew to 37.

### 2.4. Kinetic Growth Assay

The 15 synergistic interactions underwent a detailed kinetic growth study (Figures S2–S9) to analyze the behavior of each microorganism for 24 h under the circumstances cited in Section 4.5.

In general terms, we observe for all of them how the mixture of commercial ABX and CIN, when both were at their MIC_comb_, provoked a complete inhibition of the bacterial growth (Figures S2–S9). In the same way, Figures S2–S9 also illustrate the complete growth inhibition for the experiments when the natural and commercial compounds were administered individually at their respective MIC_alone_.

On the contrary, this effect is not seen when each compound at that MIC_comb_ is applied individually to the bacteria. In most of these experiments, a lower OD was measured after the lag phase when compared to the positive control.

## 3. Discussion

### 3.1. Antimicrobial Activity Analysis of Cinnamaldehyde and Commercial Antibiotics

Cinnamaldehyde is a well-studied substance with a wide range of properties. Its antimicrobial activity has been extensively evaluated. However, to the best of our knowledge, no previous studies with our identical Gram-positive and Gram-negative bacteria strains have been reported for macro- and/or microdilution assays (Table 1). The only exceptions are the works by Sim et al. [26] on *P. aeruginosa* (ATCC 27853) and *E. coli* (ATCC 25922), by Ferro et al. [28] on *P. aeruginosa* (ATCC 27853), and the research by Bianchi et al. [29] on *E. coli* (ATCC 25922). In the first publication, for the MICs for *E. coli* (157 mg/mL) and for *P. aeruginosa* (630 mg/mL), Sim et al. provided lower values than ours (see Table 1), even using the trans-isomer as we did. In the second work, the MIC obtained by Ferro et al. [28] for *P. aeruginosa* (1000 mg/mL) equals ours (Table 1). Finally, in this third article, our MIC value for *E. coli* (500 mg/mL, Table 1) doubles that obtained by Bianchi et al. (256 mg/mL) [29], which, at the same time, doubles the amount given by Sim et al. [26]. Regarding other studies, most of the antimicrobial tests are designed to evaluate the efficacy of essential oils (not isolated components) [45,46] and/or isolated CIN, but on different bacterial strains from those used in the current research.

Commercial antibiotics were also tested on the 14 bacterial strains. In Table 1, experimental values are shown together with MICs obtained by other authors. As mentioned in the Results section, only referenced MIC values for the same strain and macro or microdilution methods are given in that table. Most of our results coincide with the bibliographic ones, for example, AMO MIC on *A. baumannii* and *E. coli*, ampicillin (AMP) on *E. coli*, tetracycline clorhydrate (TC) on *P. aeruginosa*, and CHL on *B. subtilis* and *E. coli*, or they are in the same order of magnitude as the reported values, as happens with the remaining data. This was observed when the microdilution method was used in both sets of MIC values (Exp. and Lit., Table 1). However, for STM and CHL on *A. baumannii*, AMP and AMO on *P. aeruginosa*, and CHL on *S. marcescens*, MIC data differ from ours. The cause could be the fact that in these reported studies, they applied the macrodilution method instead of the microdilution one.

Different mechanisms of action for CIN have been reported, depending on the type of bacteria. Most of them include alteration of the cell membrane structure, disruption of the bacterial biofilms, or gene inhibition, as will be described in the paragraphs below.

#### 3.1.1. Gram-Positive

For *L. monocytogenes* (different strains from ours), values of trans-cinnamaldehyde MIC from 250 [47] to 512 µg/mL [48] or 640 µg/mL [49] were found, from which the last two are quite close to the MIC we report for CIN in this research (Table 1). CIN seems to reduce the swimming motility of *L. monocytogenes*, preventing the early stages of biofilm [49]. In previous studies about fungi, this microbicidal power of CIN had been attributed to the high electrophilic properties of the carbonyl group (Figure 1), which turns the compound into a very reactive structure, ready to interact with sulfhydryl and amino moieties from the microorganism proteins [50]. Perhaps similar interactions also take place with bacterial proteins.

According to García-Salinas [51] and Beáta Kerekes [47], the MIC values for *S. aureus* (a different strain from ours) were 400 µg/mL (very close to our result), although the stereochemistry of the aldehyde is not specified.

For *S. agalactiae*, a MIC for trans-cinnamaldehyde of 660 µg/mL was described, slightly higher than our result of 500 mg/mL [52].

For *E. faecalis*, authors like Ali et al. and Ferro T et al. [53,54] seem to agree with the fact that CIN inhibits bacterial biofilm formation through regulation of exopolysaccharides and/or downregulating genes related to the Quorum Sensing-Fsr system [53], whose contribution to biofilm formation is via gelatinase production. On the other hand, superoxide radicals are secreted extracellularly in large quantities by *E. faecalis* but not by the other microorganisms studied [55,56,57,58]. In our experiments on *E. faecalis* (Table 1), the MIC was slightly higher than the others, as will happen with that of *P. aeruginosa*.

Concerning the proven antimicrobial activity of CIN on *S. aureus*, one of the most interesting results is that reported by Baskaran et al. [59], who verified that the effectiveness of this bioactivity persisted for 10 days in milk infected with the cited Gram-positive bacteria. With respect to its mechanism of action, some authors found that biofilm formation is also suppressed by the natural compound [51,60], while others [51,61] suggested a cell membrane disruption or lysis of the peptidoglycan molecule and the subsequent leakage of intracellular content.

CIN was also active on *B. subtilis* (Table 1). The authors [62] explained the CIN mode of action via delocalization of membrane-associated proteins, which are involved in division and cell shape processes.

#### 3.1.2. Gram-Negative

For Gram-negative bacteria, on the contrary, it was possible to find MICs referenced in the literature for the same strains of *E. coli* and *P. aeruginosa* used in our study (Table 1). Other authors proposed MIC values for CIN ranging from 400 [61] to 519 µg/mL on different strains of the two cited bacteria [63]. The *E. coli* outer membrane has proven to be completely lysed by CIN [51]. Thanks to confocal microscopy, serious membrane damage was observed in this kind of bacteria when exposed to the natural compound. Apart from that, CIN was also able to inhibit biofilm formation [60] and, from a more clinical perspective, it also prevented bacteria from adhering to host tissues or Hep-cells [61].

Even though the other strains in our study differ from those found in the literature, trans-cinnamaldehyde has proven its antimicrobial activity for all the bacteria tested, like *A. baumannii*, with a MIC of 310 µg/mL [64], slightly lower than our result (500 µg/mL, Table 1).

For *K. pneumoniae*, our results (500 µg/mL, Table 1) agree with those found in the literature, ranging from 281 [65] to 519 µg/mL [63], although the strains are different.

For *S. enterica*, Liu et al. [48] describe MIC values of 1024 µg/mL and V.T. Nair [66] of 656 µg/mL for cinnamaldehyde. Both authors give results higher than those found in our study (500 µg/mL) (Table 1). Structural changes were also observed in *S. enterica* [25] and *K. pneumoniae* [63] when treated with CIN, while some other researchers hypothesized that the CIN carbonyl group might bind to proteins, provoking the inhibition of amino acid decarboxylase [67], modifying the production of bacteria metabolites.

For *S. marcescens*, available results (281 µg/mL) [65] are lower than our value of 500 µg/mL.

*K. aerogenes* and *P. aerogenes* have not been tested with CIN or with essential oils containing them.

No conclusive information has been found about the specific modes of action of CIN on *K. aerogenes*, *P. aerogenes*, or *S. marcescens*.

On the contrary, *P. aeruginosa* has been extensively studied. According to Didehdar et al., CIN inhibits PA01 biofilm formation via quorum sensing (QS) inhibition because the compound represses lasB, rhlA, and pqsA genes [60]. There are also some important findings about the use of CIN sub-inhibitory concentrations in this bacteria [28], i.e., doses lower than the MIC. The first of them is that the natural compound did not induce an adaptative phenotype in *P. aeruginosa*, *E. faecalis,* or *S. aureus* [28], suggesting that CIN may not have generated resistant strains in the time studied. In addition, at these lower proportions, CIN diminishes the bacteria’s metabolic rate [28]. Finally, the compound inhibits biofilm formation [28].

### 3.2. Assessment of the Combination

Combining antibiotic therapies is a strategy often employed in the treatment of multidrug-resistant infections. It has previously been utilized for the most resistant Gram-negative infections, but evidence suggests that coadministration of two antimicrobial drugs can be one of the cornerstones for the treatment of Gram-positive infections in the future as well [68]. Our results (Table 1) prove that the antimicrobial activity of cinnamaldehyde and, therefore, the feasibility of using this natural compound in combination with commercial antibiotics lead to a decrease in their MIC. Effects resulting from the simultaneous use of antibiotics with different plant extracts have been studied by a number of researchers [69,70,71], but less attention has been previously paid to the synergistic effects of an individual commercial antibiotic and a natural component, in particular, trans-cinnamaldehyde.

Although the mechanisms of action of the synergies may be different depending on the antibiotic or bacterium, they all seem to draw attention to the fact that damaging the cell envelope by CIN facilitates the entry of ABXs into it, making them more effective. On the other hand, the action of CIN on the membrane might also alter microbial resistance mechanisms against ABXs, such as efflux pumps, preventing them from ejecting ABXs out of the cell [72,73].

#### 3.2.1. Beta-Lactams

Combinations of CIN and beta-lactams used in this study have only been described for AMP by Palaniappan et al. [25]. Although they used resistant strains, their work shows synergy among AMP and CIN against *S. enterica*, *S. aureus*, and *E. coil* [25]. However, no studies are offered for the combination with AMO.

In our study, CIN interactions with beta-lactams gave better results than those with aminoglycosides in Gram-negative bacteria. AMP and AMO interactions resulted in synergy in *P. aerogenes* (Figure S7a,b) and, additionally, AMP synergized with CIN in *K. pneumoniae* and *L. monocytogenes* (Figures S3b and S6). In addition, CIN achieved a MIC reduction of AMO up to 94% in *P. aerogenes* (Table 2).

Beta-lactam antibiotics are relatively small and hydrophilic molecules, but the hydrophobic outer membrane architecture of Gram-negative bacteria protects them from hydrophilic antibiotics. Porins have been shown to be important entrance points for antibacterial chemicals in these species [74].

Literature reports that beta-lactams inhibit the activity of PBP (penicillin-binding proteins) enzymes that are needed for the cross-linking of peptidoglycans during the final step in cell wall biosynthesis [75,76]. This causes the cell wall to weaken due to fewer cross-links, altering the correct osmotic gradient of the cell [76].

This effect of the ABXs might be added to that of CIN to disrupt the cell membrane by changing the gradient of the electric cations, thus provoking the cell to burst in the end. One of the resistance mechanisms that bacteria can develop against beta-lactams is efflux pumps, so coadministration with cinnamaldehyde might prevent the intracellular antibiotic concentration from a sudden reduction [77]. This interaction was already described by Karumathil et al. for trans-cinnamaldehyde in *A. baumannii* [72].

#### 3.2.2. Aminoglycosides

Many of the interactions among aminoglycosides and CIN resulted in FIC values < 0.5 (Table 2) and, therefore, synergy. A synergistic combination between STM and CIN was already described by Liu et al. [48] against foodborne pathogens like *L. monocytogenes* and *S. enterica*, both included in our study (Table 2). Their results are consistent with our observations, with FIC values of 0.5 and 0.37, respectively, both with a 75% reduction of ABX MIC (Table 2). Combinations with gentamycin (GTM) were also noted by Wang et al. against *S. aureus* in clinical strains with a range of FICs between 0.37 and 0.5 [73], while our work also shows a clear synergy interaction with a FIC value of 0.19 (Table 2). For the Gram-positive *S. agalactiae*, *E. faecalis*, and *S. aureus*, both aminoglycosides interacted synergistically (Table 2). Jatin Chadha et al. described a synergistic activity among GTM and *P. aeruginosa*, which, in our case, resulted in a FIC index of 1.5 and, therefore, addition [78], maybe because the strain was different. On the other hand, our results are consistent with those of Thirapanmethee et al., who described additive effects among GTM and CIN against five strains of *A. baumannii* [11]. The most successful antibiotic group interactions are found in aminoglycosides for Gram-positive microorganisms: *E. faecalis*, *L. monocytogenes*, *S. agalactiae*, and *S. aureus* (Table 2). Especially relevant is the experiment where CIN reduced the MIC of STM against *S. aureus* by 94%.

Aminoglycosides mechanism of action involves protein synthesis inhibition by altering the 30 s subunit of the prokaryotic ribosome [75,79]. These antibiotics are large, polar molecules that cannot passively diffuse through the bacterial cell envelope, so they require active transport systems to enter the bacterial cell [80]. If the cell membrane is disrupted by CIN, the antibiotic entry rate into the cytosol will increase, and the intracellular concentration of the antibiotic to inhibit protein synthesis will be higher compared to the treatment when the natural substance is absent [68]. Furthermore, the interaction with CIN that can destabilize the cell envelope could also neutralize one of the main mechanisms bacteria employ to remove antibiotics, the efflux pumps, maintaining the concentration of ABXs in the cytoplasm [81].

#### 3.2.3. Macrolides and Amphenicoles

Synergy between macrolides like ERY and CIN was detailed by Palaniappan et al. [25] for *S. enterica* and *E. coli*. On the contrary, an additive interaction was observed in both of our tests, but while on *E. coli* no ABX MIC reduction was seen, a reduction of 94% was measured on *S. enterica*. Additionally, synergistic interactions against clinical strains of *E. coli* were described by Visvalingam et al. [82] when combining this macrolide with CIN. The variations in the FIC values (between 0.3 and 0.5) when compared to ours may be attributed to the differences in the strains tested. However, our findings do concur with those from Halim Topa et al. [27], who investigated the same combination against *P. aeruginosa* biofilms and found additive action like we did.

Macrolides, like ERY, and amphenicols, like CHL, share a similar mechanism of action by inhibiting the elongation of the protein chain at the ribosomal unit [75,83]. They both are broad-spectrum antibiotics that are effective against a wide range of bacteria, and they both enter the bacteria through passive diffusion [84]. Furthermore, they both synergized with CIN on *S. marcescens* (Figure S9a,b), a Gram-negative bacterium, in which CIN achieved an ABX MIC reduction of 75% for ERY and 98% for CHL, the highest obtained in this research (Table 2). In addition, CHL combination with CIN also resulted in synergy for the Gram-positive *S. agalactiae* (Figure S4), with an ABX MIC reduction of 75% (Table 2).

CHL and ERY are both lipophilic molecules that enter the bacterial cell through passive diffusion across the cell membrane. CIN membrane disruption could be the mechanism in both ABXs synergies with CIN, allowing greater entry of both antibiotics, which would act on its ribosomal target [75].

When studying the MIC reduction (%) (Table 2), it can be observed that CIN helped reduce CHL MIC in many other cases that are considered additive, for example, *A. baumannii* and *K. pneumoniae*.

Natural products have also been seen to induce the expression of efflux pumps in bacteria. Its induction can result in decreased susceptibility of bacteria to antibiotics that target the ribosome [85]. Cinnamaldehyde has also been studied as an efflux pump inductor for *P. aeruginosa* [86]. *P. aeruginosa*, together with *K. aerogenes*, *A. baumannii*, and *E. coli*, are the only bacteria for which synergies have not been observed, and all of them are especially prone to generating resistance. It should be noted that every bacterial strain has an individual behavior responding to the damage that the drug can cause, and therefore each combination should be studied separately.

When analyzing candidate molecules to be used as antibiotics, attention must also be paid to their physicochemical properties. Some of them are extremely related to their potential bactericidal activity, such as molecular weight, partition coefficient, hydrogen bonding, and solubility [87]. Antimicrobials with a low molecular weight, like CIN (132.16 g/mol) [88], usually target better Gram-negative cultures [6]. CIN has a LogP of 1.90, which means it is slightly more lipophilic than hydrophilic [88]. Finally, water solubility is a crucial feature in order to test in vitro antibacterial activity. CIN is fairly insoluble in water (1.42 mg/mL at 25 °C), and therefore, the need for an organic solvent rises [89]. Pure organic solvents may be harmful to bacteria or alter their growth. That is why a toxicity test (Supporting Information) was conducted to determine whether the concentrations needed to dissolve CIN have intrinsic damaging activity on the bacterium culture.

Analysis of antibiotic interactions (Figures S2–S9) shows how the antimicrobial hypothesis of cell disruption might fit in. Many authors believe that naturally occurring bioactive substances primarily target the cell membrane and are more effective against Gram-positive bacteria [90]. CIN activity has been shown to affect not only Gram-positives but Gram-negatives at the same level, which, in fact, shows that, at least, one of the possible modes of action is unspecific, like cell disruption. Furthermore, its slight lipophilicity and low molecular weight would enable CIN to penetrate the lipidic membranes and exert some other mechanisms.

### 3.3. Kinetics

As it was reported in the Results section, when synergies were observed, the combination of the commercial ABX and CIN at their respective MIC_comb_ resulted in a complete inhibition of bacterial growth with time (Figures S2–S9). However, if tested alone at these same concentrations, complete inhibition was never achieved because these doses are actually sub-inhibitory (all of them are below their respective MIC_alone_). If we focus on ABXs kinetics when tested alone at their MIC_comb_ on the different microorganisms, the general trend is an increase in their lag phase when compared to that of the control, regardless of whether they are Gram-positive or negative (Figures S3, S4, S5b, S6, S7b, S8 and S9a), although the effect is stronger on Gram-negative. So is the effect of CIN for all tested bacteria, except for *K. pneumoniae* (Figure S6) and *S. marcescens* (Figure S9), whose lag phase is approximately the same. The lag phase is the moment when cells are adapting to the new environment, and this adaptation can be slowed down if the situation is hostile [91]. According to our data, CIN provoked a longer lag phase than ABXs in the following situations: *S. aureus*-STM (Figure S5a), *P. aerogenes*-AMO (Figure S7a), *P. aerogenes*-AMP (Figure S7b), and *S. enterica*-STM (Figure S8), which could mean that these bacteria perceive the environment with CIN more aggressively than that caused by the commercial drug.

Most antibiotics demonstrated a reduction in the growth rate in the exponential phase (in other words, a diminution in the slope in the log phase) when added alone at their respective MIC_comb_. This step in bacterial growth corresponds to the moment in which bacteria are reproducing but also to a stage when they become bigger because of their active metabolism [91]. This inhibitory effect is especially pronounced on *E. faecalis*-STM, GTM, and CIN (Figure S2a,b), *L. monocytogenes*-STM (Figure S3a), *S. agalactiae*-GTM, STM, and CHL (Figure S4), *S. aureus*-STM and CIN (Figure S5a), *K. pneumoniae*-AMP (Figure S6), *S. enterica*-CIN (Figure S8), and *S. marcescens*-CHL (Figure S9b). If the slopes of the log phase of experiments with ABXs at their MIC_comb_ are compared to the respective slopes of the log phase of assays with CIN at their MIC_comb_, the trend seems to be that ABX slopes are lower than CIN slopes (Figures S3, S4, S6 and S9b). Only for experiments on *E. faecalis* (Figure S2) and *S. enterica* (Figure S8) was the opposite behavior found. For the remaining tests, no difference is highlighted. A higher slope would mean a higher reproduction rate and/or a larger cell size.

The stationary phase was reached in all experiments for the control (Figures S2–S9). Nonetheless, this did not happen for *E. faecalis*-STM (Figure S2a), *L. monocytogenes*-STM and AMP (Figure S3), *S. agalactiae*-CHL (Figure S4c), *S. aureus*-CIN (Figure S5a), *K. pneumoniae*-AMP (Figure S6), and *P. aerogenes*-AMP and CIN (Figure S7b) at their respective MIC_comb_. The absorbance of these stationary phases was always lower than that of the control except for the following experiments: *S. aureus*-CIN (Figure S5b), *K. pneumoniae*-CIN (Figure S6) and *P. aerogenes*-AMO (Figure S7a).

## 4. Materials and Methods

### 4.1. Bacterial Strains

The strains used for this research were the Gram-positive bacteria *Staphylococcus aureus* (ATCC 9144), *Streptococcus agalactiae* (ATCC 12386), *Enterococcus faecalis* (ATCC 19433), *Bacillus subtilis* (ATCC 6633), and Gram-negative *Listeria monocytogenes* (ATCC 7644), *Escherichia coli* (ATCC 25922), *Salmonella enterica* (ATCC 13311), *Proteus mirabilis* (ATCC 35659), *Pseudomonas aeruginosa* (ATCC 27853), *Acinetobacter baumannii* (ATCC 19606), *Klebsiella aerogenes* (ATCC 13048), *Klebsiella pneumoniae* (C6), *Serratia marcescens* (ATCC 13880), and *Pasteurella aerogenes* (ATCC 27883). Strains were acquired and lyophilized in culti-loops (Thermo Scientific, Waltham, MA, USA). To avoid mutations in the initial strains, they were frozen in Cryoinstant Mix cryotubes (Deltalab, Barcelona, Spain), following the manufacturer’s instructions. Then, it was stored at −80 °C (Froilabo, Collégien, France, Trust −80 °C) and rehydrated for its use following the technical information sheet of each microorganism. Culture, medium, and test conditions for each microorganism optimal growth and assay success were performed according to specifications described in the technical sheet of each strain (www.atcc.org, accessed on 12 January 2023).

Prior to the use of any microorganism, the bacterial inoculum was re-cultured from the cryotubes and incubated (J. P. Selecta) at the conditions required for each optimum bacterial growth for 24 h. Then, the overnight culture was adjusted to the required bacterial optical density of 0.5 McFarland scale, corresponding to 2.5 × 10^8^ CFU/mL [30].

### 4.2. Compounds to Be Tested and Stock Solutions

CIN and seven commonly used antibiotics (ABXs) were used as antimicrobials. The CAS numbers and purity information of these compounds and trans-cinnamaldehyde are shown in Table 3.

ABXs were our antimicrobial reference agents: AMP, AMO, GTM, STM, ERY, TC, and CHL. All of them were soluble in sterile water, and no other solvents were used.

CIN was dissolved in sterile water with 5% of DMSO (Table 3) at a final concentration of 4000 μg/mL (stock solution). This concentration of DMSO was the minimum concentration required for the trans-cinnamaldehyde to be dissolved in an aqueous solution. Sterile conditions were ensured for all operations. The tested concentrations of ABXs and CIN ranged from 0.1 to 2000 μg/mL.

### 4.3. Toxicity and Antimicrobial Susceptibility Tests

The minimum inhibitory concentration (MIC) and the minimum bactericide concentration (MBC) of CIN and the MIC of ABXs were determined. In order to ensure that the solvent DMSO was innocuous (at the concentration used) for each bacterial strain, toxicity tests were previously performed.

The procedure to develop these tests was the same as that for the determination of the MIC (the lowest concentration of a substance that completely inhibits bacterial growth).

The MIC of all antimicrobial agents against the bacterial strains was performed by the standard broth microdilution method according to the Clinical and Laboratory Standards Institute [30,33,92]. Each experiment was carried out in triplicate. Briefly, 100 μL of broth were added to each well of the 96-well plate (round bottom), followed by 100 μL of antimicrobial (or DMSO) solution. Samples were tested in two-fold dilutions, followed by the addition of 10 μL of the bacterial suspension once it was adjusted according to the McFarland scale, as previously described. Positive and negative controls were also added. The positive control measures the standard growth of bacteria with no antimicrobial agent. Negative control involved only culture broth to ensure that there was no microbial growth or contamination in it. After the incubation period (24 h), absorbance was measured at each bacteria’s optimum temperature and at 625 nm with a BioTek™ Synergy H1 Hybrid multimode microplate reader.

DMSO toxicity results are given as a percentage of survival (Survival (%)), calculated as follows (Equation (1)):

Survival (%) = (Abs_DMSO_)·100/Abs_C_ (1)

where Abs_DMSO_ is the average of the absorbance of the wells with DMSO solution at a given concentration and Abs_C_ is the average of the absorbance of the positive control wells. The doses tested for DMSO ranged from 0.16 to 20%, and it was considered non-toxic when the survival was higher than 99%. Experiments were performed in triplicate. Survival data are expressed as the mean ± standard deviation.

The MBC was defined as the lowest concentration that yielded no colony growth by subculturing on agar plates. It was tested only for CIN. Therefore, aliquots from different wells from the resulting MIC and subsequent wells with higher concentrations were cultured and incubated for 24 h [93].

### 4.4. Checkerboard Assay

After the identification of MIC and MBC, the possible interactions between the natural compound and each commercial antibiotic were studied. Combination tests were performed using the checkerboard assay method [94,95,96].

New stock solutions of each antimicrobial agent were prepared. The concentration of each stock solution was 4 times its reported MIC, which had been obtained for each microorganism individually beforehand. Trans-cinnamaldehyde serial two-fold dilutions were added from the 1st to the 7th column of the 96-well plate (round bottom), while antibiotic solutions were added from rows A to G. Therefore, each well resulted in a different concentration of both antimicrobial agents tested. The most concentrated well was A1, while the least concentrated one resulted in G7. Once the serial two-fold dilutions were applied to the plate, 10 μL of bacteria inoculum were added after its adjustment to the right McFarland concentration, as previously described. Positive (bacteria without antimicrobial) and negative (culture medium without bacteria) controls were also prepared [97].

Two sorts of Fractional Inhibitory Indices (FIC) were obtained to evaluate the interactions: FIC A and FIC B (Equations (2) and (3), respectively).

(2) $$FIC\;A=\frac{MIC\;of\;A\;in\;combination\;with\;B}{MIC\;of\;A\;alone}$$

(3) $$FIC\;B=\frac{MIC\;of\;B\;in\;combination\;with\;A}{MIC\;of\;B\;alone}$$

With them, the ΣFIC, as the addition of both FIC A and FIC B, was calculated, and, according to the resulting value, the type of interaction between the two compounds was established. The interactions were considered synergistic if ΣFIC < 0.5; additive if 0.5 < ΣFIC < 4; and antagonistic if ΣFIC > 4 [98,99,100].

Experiments were performed in triplicate and with flow chamber sterility (Model MSC Advantage 1.2).

### 4.5. Kinetic Growth Assay

The methodology followed was described by the Clinical and Laboratory Standards Institute with slight modifications [78,101].

On a 96-well plate (round bottom), wells were filled with: (i) commercial antibiotic solution at its MIC when tested alone (MIC_alone_), (ii) commercial antibiotic solution at its MIC when tested in combination with the natural product (MIC_comb_), (iii) natural compound solution at its MIC when tested alone, (iv) natural product solution at its MIC when tested in combination with the commercial antibiotic, and (v) solution made of natural compound and commercial antibiotic both at their respective MICs when tested in combination [102].

The absorbance was measured every hour for 24 h at 625 nm with the SPECTROstar Nano of BMG Labtech at each bacterial optimal temperature. Experiments were performed in triplicate; all data were expressed as mean ± standard deviation.

## 5. Conclusions

One of the key strategies to counteract multidrug resistance pathogens is the coadministration of two active molecules with complementary modes of action, where one of them is a commercial antibiotic and the other could be, for example, a natural product with confirmed antimicrobial properties, like trans-cinnamaldehyde. If the substances act in a synergistic way, the action of their combination is more powerful than that of the compounds when added separately, leading to the use of lower concentrations of these substances to get the same effect.

In this paper, the antimicrobial properties of trans-cinnamaldehyde were studied on 14 different Gram-positive and Gram-negative bacteria. The MIC for seven commercial antibiotics (AMP, AMO, STM, GTM, ERY, TC, and CHL) was also determined. MIC values for the natural product were 500 mg/mL for all bacteria except for *E. faecalis* and *P. aeruginosa*, whose values were 1000 mg/mL. Antibiotic MICs equaled literature values only when the strain and the method were exactly the same as ours.

Then, the combination of CIN with each antibiotic was applied to every bacterium. This study led to 15 synergistic interactions (on *E. faecalis*, *L. monocytogenes*, *S. agalactiae*, *S. aureus*, *K. pneumoniae*, *P. aerogenes*, and *S. enterica*) in which the MIC of the commercial antibiotic was reduced up to 75% in the worst synergy and up to 98% in the best. One-third of the synergistic interactions were shown for STM. The remaining synergies were observed for GTM, AMP, CHL, AMO, and ERY. Nevertheless, some of the additive interactions also reported MIC antibiotic reductions (ranging from 50 to 94%). Considering this fact, the number of interactions that provoked an antibiotic MIC reduction increased to 37. Therefore, if one of the possible solutions to reduce antibiotic resistance consists of reducing the antibiotic dose, additive combinations may also be contemplated for future work.

Only the 15 synergistic combinations underwent the kinetic study of the bacterial growth, where the synergistic effect of the combination was perfectly clear and confirmed the results obtained when the checkboard assay was previously applied.

According to our experimental data and previous research, the theory we support is that the main synergistic mechanism of CIN when added with commercial antibiotics is the disruption of the bacterial envelope, which would alter its permeability, helping the commercial antibiotic enter the cell and inhibit its growth. This would not mean that other mechanisms, such as efflux-pump inhibition or gene expression inhibition, do not take place, but they would be complementary or secondary modes of action of the CIN synergistic effects.

Polyphenols, like trans-cinnamaldehyde, are widely available bioactive compounds that are not only common but also considered safe for human consumption by the FDA and the European Council. CIN has been probed to show antimicrobial properties against pathogenic bacteria and has demonstrated synergistic behavior when combined with commercial antibiotics. Although the mechanism of action is not completely elucidated, this natural compound deserves much more attention as a candidate for future antimicrobial therapies because, in combination with commercial antibiotics, the dose reduction of the latest can reach extremely high values.

## Figures and Tables

**Table 1:** Minimum inhibitor concentration (MIC_alone_ (mg/mL)) of commercial antibiotics and cinnamaldehyde tested alone on the selected bacteria.

MIC_alone_ (μg/mL)									
Bacteria		AMP	AMO	STM	GTM	ERY	TC	CHL	CIN
*Bacillus subtilis* (ATCC 6633)	Exp.	0.3	0.3	8	8	<0.5	5	1.5	500
	Lit.	1 ^f^		1.56 ^j^			1.562 ^n^	1.56 ^j^	
*Enterococcus faecalis* (ATCC 19433)	Exp.	500	500	250	25	100	12.5	125	1000
	Lit.								
*Listeria monocytogenes* (ATCC 7644)	Exp.	125	62.5	200	12.5	125	12.5	10	500
	Lit.							64 ^o^	
*Streptococcus agalactiae* (ATCC 12386)	Exp.	62.5	62.5	100	18.75	125	0.19	50	500
	Lit.								
*Staphylococcus aureus* (ATCC 9144)	Exp.	0.15	0.625	100	25	0.62	12.5	30	500
	Lit.								
*Acinetobacter baumannii* (ATCC 19606)	Exp.	125	62.5	75	25	10	0.75	62.5	500
	Lit.		64 ^g^	256 ^k^	16 ^g^			256 ^p^	
*Escherichia coli* (ATCC 25922)	Exp.	7.5	7.5	15.65	15.65	62.5	0.75	7.5	500
	Lit.	7.5 ^e^	2–8 ^h^	32 ^l^	64 ^m^	32 ^a^		7.5 ^e^	256 ^d^
									157 ^b^
*Klebsiella aerogenes* (ATCC 13048)	Exp.	1500	1000	4.5	2.5	31.25	1.9	10	500
	Lit.							8 ^q^	
*Klebsiella pneumoniae* (C6)	Exp.	500	125	10	0.78	62.5	1.17	15	500
	Lit.								
*Pasteurella aerogenes* (ATCC 27883)	Exp.	125	500	15.6	6.25	62.5	10	8	500
	Lit.								
*Pseudomonas aeruginosa* (ATCC 27853)	Exp.	1000	>1000	62.5	12.5	500	25	250	1000
	Lit.	>64 ^b^	0.12 ^i^	12.5 ^j^	0.5–2 ^e^		8–32 ^e^	100 ^r^	1000 ^c^
									630 ^b^
*Salmonella enterica* (ATCC 13311)	Exp.	<0.15	<0.15	50	0.75	31.25	1.17	3.9	500
	Lit.								
*Serratia marcescens* (ATCC 13880)	Exp.	125	125	0.4	5	250	250	125	500
	Lit.							6.25 ^s^	
*Proteus mirabilis* (ATCC 35659)	Exp.	0.08	0.62	12.5	25	<0.08	0.08	7.8	-
	Lit.								

MICs were obtained by applying the microdilution method. Experimental data (Exp.) are shown at the top of every cell, while data extracted from literature (Lit.) for the same strain and both micro and/or macrodilution methods are given at the bottom. Only bibliographic data related to the same strains as the ones used in this work was considered in this table. (-): not tested. ^a^ Ref. [3]; ^b^ Ref. [26]; ^c^ Ref. [28]; ^d^ Ref. [29]; ^e^ Ref. [30]; ^f^ Ref. [31]; ^g^ Ref. [32]; ^h^ Ref. [33]; ^i^ Ref. [34]; ^j^ Ref. [35]; ^k^ Ref. [36]; ^l^ Ref. [37]; ^m^ Ref. [38]; ^n^ Ref. [39]; ^o^ Ref. [40]; ^p^ Ref. [41]; ^q^ Ref. [42]; ^r^ Ref. [43]; ^s^ Ref. [44].

**Table 2:** MICs for commercial antibiotics and cinnamaldehyde when tested in combination (MICcomb (μg/mL)), FIC, ΣFIC, the subsequent type of activity for every combination bacteria-natural compound-commercial antibiotic, and MIC reduction (%) * for each compound. Synergies are highlighted in pale gray. Antibiotic reductions ≥ 50% are highlighted in dark gray.

Gram+/−	Bacteria	Compounds	MIC_comb_ (mg/mL)	FIC	ΣFIC	Activity	MIC Reduction (%)
GRAM+	*E. faecalis*	CIN	250	0.25	0.5	Synergy	75
		STM	62.5	0.25			75
		CIN	250	0.25	0.37		75
		GTM	3	0.12			88
		CIN	500	0.5	0.75	Addition	50
		AMP	125	0.25			75
		CIN	500	0.5	0.75		50
		AMO	125	0.25			75
		CIN	500	0.5	1.5		50
		ERY	100	1			0
		CIN	500	0.5	2.5		50
		TC	25	2			−100
		CIN	500	0.5	4.5	Antagonism	50
		CHL	500	4			−300
	*L. monocytogenes*	CIN	125	0.25	0.5	Synergy	75
		AMP	31.25	0.25			75
		CIN	125	0.25	0.5		75
		STM	50	0.25			75
		CIN	250	0.5	2.5	Addition	50
		AMO	125	2			−100
		CIN	250	0.5	0.62		50
		GTM	1.56	0.12			88
		CIN	250	0.5	1		50
		ERY	62.5	0.5			50
		CIN	250	0.5	1		50
		TC	6.25	0.5			50
		CIN	250	0.5	8.5	Antagonism	50
		CHL	62.5	8			−525
	*S. agalactiae*	CIN	125	0.25	0.5	Synergy	75
		STM	25	0.25			75
		CIN	125	0.12	0.37		88
		GTM	4.6	0.25			75
		CIN	31.5	0.25	0.5		75
		CHL	12.5	0.25			75
		CIN	250	0.5	1	Addition	50
		AMP	31.25	0.5			50
		CIN	250	0.5	1		50
		AMO	31.25	0.5			50
		CIN	250	0.5	1		50
		ERY	62.5	0.5			50
	*S. aureus*	CIN	125	0.25	0.31	Synergy	75
		STM	6.25	0.06			94
		CIN	31.25	0.06	0.19		94
		GTM	3	0.13			88
		CIN	250	0.5	1.5	Addition	50
		TC	12.5	1			0
		CIN	500	1	>3		0
		CHL	>60	2			<−100
GRAM−	*A. baumannii*	CIN	125	0.5	0.75	Addition	75
		AMP	62.5	0.25			50
		CIN	500	1	2		0
		AMO	62.5	1			0
		CIN	250	0.5	1.5		50
		ERY	10	1			0
		CIN	250	0.5	1		50
		CHL	31.25	0.5			50
		CIN	125	0.25	>2.25		75
		GTM	>50	2			<−100
		CIN	250	0.5	4.5	Antagonism	50
		STM	300	4			−300
	*E. coli*	CIN	250	0.5	0.75	Addition	50
		STM	3.9	0.25			75
		CIN	250	0.5	0.75		50
		GTM	3.9	0.25			75
		CIN	250	0.5	1.5		50
		ERY	62.5	1			0
	*K. aerogenes*	CIN	250	0.5	0.62	Addition	50
		AMP	187.5	0.12			87
		CIN	250	0.5	0.62		50
		AMO	125	0.12			87
		CIN	250	0.5	2.5		50
		ERY	62.5	2			−100
		CIN	250	0.5	8.5	Antagonism	50
		CHL	80	8			−700
	*K. pneumoniae*	CIN	125	0.25	0.5	Synergy	75
		AMP	125	0.25			75
		CIN	250	0.5	2.5	Addition	50
		AMO	250	2			−100
		CIN	250	0.5	1		50
		STM	5	0.5			50
		CIN	125	0.25	2.5		75
		ERY	125	2			−100
		CIN	250	0.5	1		50
		CHL	7.5	0.5			50
	*P. aerogenes*	CIN	125	0.25	0.5	Synergy	75
		AMP	31.25	0.25			75
		CIN	62.5	0.13	0.19		87
		AMO	31.25	0.06			94
		CIN	250	0.5	1.5	Addition	50
		STM	15.6	1			0
		CIN	250	0.5	1.5		50
		ERY	62.5	1			0
		CIN	250	0.5	1.5		50
		TC	10	1			0
	*P. aeruginosa*	CIN	500	0.5	0.62	Addition	50
		AMP	125	0.12			87
		CIN	250	0.25	0.75		75
		STM	31.25	0.5			50
		CIN	250	0.25	1.5		75
		GTM	12.5	1			0
		CIN	500	0.5	1.5		50
		ERY	500	1			0
		CIN	500	0.5	1.5		50
		TC	25	1			0
		CIN	500	0.5	4.5	Antagonism	50
		CHL	1	4			−300
	*S. enterica*	CIN	62.5	0.12	0.37	Synergy	87
		STM	12.5	0.25			75
		CIN	250	0.5	0.56	Addition	50
		ERY	1.9	0.06			94
	*S. marcescens*	CIN	125	0.25	0.26	Synergy	75
		CHL	1.9	0.01			98
		CIN	125	0.25	0.5		75
		ERY	62.5	0.25			75
		CIN	250	0.5	1	Addition	50
		AMP	62.5	0.5			50
		CIN	250	0.5	1		50
		AMO	62.5	0.5			50
		CIN	250	0.5	0.62		50
		TC	31.25	0.12			87

* MIC reduction (%) = (MIC_alone_ − MIC _comb_) × 100/MIC_alone_.

**Table 3:** Name, CAS number, provider, and purity of the compounds tested.

Antibiotic	CAS	Provider	Purity/%
Ampicillin	69-53-4	Sigma Aldrich (St. Louis, MO, USA)	>96
Amoxicillin	26787-78-0		
Gentamycin (sulphate)	1405-41-0	Acofarma (Barcelona, Spain)	
Streptomycin (sulphate)	3810-74-0		
Erythromycin	114-07-8		97.5
Tetracycline (clorhidrate)	64-75-5		99.2
Chloramphenicol	56-75-7		99.6
Trans-cinnamaldehyde	104-55-2	Sigma Aldrich	>95
DMSO	67-68-5	Fisher Bioreagents (Madrid, Spain)	>99.7

## Data Availability

All data generated or analyzed during this study are included in this manuscript and its supplementary information files.

## Supplementary Materials

The following supporting information can be downloaded at: https://www.mdpi.com/article/10.3390/plants13020192/s1. Figure S1. Survival (%) of a) Gram (-) bacteria and b) Gram (+) bacteria (B) tested in this work when exposed to different dilutions of DMSO. Figure S2. Kinetic study for cinnamaldehyde (CIN) and (a) streptomycine (STM) or (b) gentamicin (GTM) on *E. faecalis* (OD at 625 nm vs. time (h)). C+: curve for positive control. MIC CINalone and MIC ABXalone are the curves for CIN and the specific ABX, respectively, when each of them was tested alone at their respective MIC. MIC ABXcomb is the curve for the specific ABX tested alone but added at its MIC when this and CIN were tested simultaneously. MIC CINcomb is the curve for CIN tested alone but added at its MIC when this and the specific ABX were tested simultaneously. (MIC ABXcomb + MIC CINcomb) is the cruve for the combination of the mixture of the specific ABX and CIN when tested simultaneously at their respective MICs in combination. Data are given as mean ± standard deviation. Figure S3. Kinetic study for cinnamaldehyde (CIN) and (a) streptomycine (STM) or (b) ampicillin (AMP) on *L. monocytogenes* (OD at 625 nm vs. time (h)). C+: curve for positive control. MIC CIN_alone_ and MIC ABX_alone_ are the curves for CIN and the specific ABX, respectively, when each of them was tested alone at their respective MIC. MIC ABX_comb_ is the curve for the specific ABX tested alone but added at its MIC when this and CIN were tested simultaneously. MIC CIN_comb_ is the curve for CIN tested alone but added at its MIC when this and the specific ABX were tested simultaneously. (MIC ABX_comb_ + MIC CIN_comb_) is the cruve for the combination of the mixture of the specific ABX and CIN when tested simultaneously at their respective MICs in combination. Data are given as mean ± standard deviation. Figure S4. Kinetic study for cinnamaldehyde (CIN) and (a) gentamicin (GTM), (b) streptomycin (STP), or (c) chloramphenicol (CHL) on *S. agalactiae* (OD at 625 nm vs. time (h)). C+: curve for positive control. MIC CIN_alone_ and MIC ABX_alone_ are the curves for CIN and the specific ABX, respectively, when each of them was tested alone at their respective MIC. MIC ABX_comb_ is the curve for the specific ABX tested alone but added at its MIC when this and CIN were tested simultaneously. MIC CIN_comb_ is the curve for CIN tested alone but added at its MIC when this and the specific ABX were tested simultaneously. (MIC ABX_comb_ + MIC CIN_comb_) is the cruve for the combination of the mixture of the specific ABX and CIN when tested simultaneously at their respective MICs in combination. Data are given as mean ± standard deviation. Figure S5. Kinetic study for cinnamaldehyde (CIN) and (a) streptomycine (STM) or (b) gentamicin (GTM) on *S. aureus* (OD at 625 nm vs. time (h)). C+: curve for positive control. MIC CIN_alone_ and MIC ABX_alone_ are the curves for CIN and the specific ABX, respectively, when each of them was tested alone at their respective MIC. MIC ABX_comb_ is the curve for the specific ABX tested alone but added at its MIC when this and CIN were tested simultaneously. MIC CIN_comb_ is the curve for CIN tested alone but added at its MIC when this and the specific ABX were tested simultaneously. (MIC ABX_comb_ + MIC CIN_comb_) is the cruve for the combination of the mixture of the specific ABX and CIN when tested simultaneously at their respective MICs in combination. Data are given as mean ± standard deviation. Figure S6. Kinetic study for cinnamaldehyde (CIN) and ampicillin (AMP) on *K. pneumoniae* (OD at 625 nm vs. time (h)). C+: curve for positive control. MIC CINalone and MIC ABXalone are the curves for CIN and the specific ABX, respectively, when each of them was tested alone at their respective MIC. MIC ABXcomb is the curve for the specific ABX tested alone but added at its MIC when this and CIN were tested simultaneously. MIC CINcomb is the curve for CIN tested alone but added at its MIC when this and the specific ABX were tested simultaneously. (MIC ABXcomb + MIC CINcomb) is the cruve for the combination of the mixture of the specific ABX and CIN when tested simultaneously at their respective MICs in combination. Data are given as mean ± standard deviation. Figure S7. Kinetic study for cinnamaldehyde (CIN) and (a) amoxicillin (AMO) or (b) ampicillin (AMP) on *P. aerogenes* (OD at 625 nm vs. time (h)). C+: curve for positive control. MIC CIN_alone_ and MIC ABX_alone_ are the curves for CIN and the specific ABX, respectively, when each of them was tested alone at their respective MIC. MIC ABX_comb_ is the curve for the specific ABX tested alone but added at its MIC when this and CIN were tested simultaneously. MIC CIN_comb_ is the curve for CIN tested alone but added at its MIC when this and the specific ABX were tested simultaneously. (MIC ABX_comb_ + MIC CIN_comb_) is the cruve for the combination of the mixture of the specific ABX and CIN when tested simultaneously at their respective MICs in combination. Data are given as mean ± standard deviation. Figure S8. Kinetic study for cinnamaldehyde (CIN) and streptomycine (STM) on *S. enterica* (OD at 625 nm vs. time (h)). C+: curve for positive control. MIC CINalone and MIC ABXalone are the curves for CIN and the specific ABX, respectively, when each of them was tested alone at their respective MIC. MIC ABXcomb is the curve for the specific ABX tested alone but added at its MIC when this and CIN were tested simultaneously. MIC CINcomb is the curve for CIN tested alone but added at its MIC when this and the specific ABX were tested simultaneously. (MIC ABXcomb + MIC CINcomb) is the cruve for the combination of the mixture of the specific ABX and CIN when tested simultaneously at their respective MICs in combination. Data are given as mean ± standard deviation. Figure S9. Kinetic study for cinnamaldehyde (CIN) and (a) erythromycin (ERY) or (b) chloramphenicol (CHL) on *S. marcescens* (OD at 625 nm vs. time (h)). C+: curve for positive control. MIC CIN_alone_ and MIC ABX_alone_ are the curves for CIN and the specific ABX, respectively, when each of them was tested alone at their respective MIC. MIC ABX_comb_ is the curve for the specific ABX tested alone but added at its MIC when this and CIN were tested simultaneously. MIC CIN_comb_ is the curve for CIN tested alone but added at its MIC when this and the specific ABX were tested simultaneously. (MIC ABX_comb_ + MIC CIN_comb_) is the cruve for the combination of the mixture of the specific ABX and CIN when tested simultaneously at their respective MICs in combination. Data are given as mean ± standard deviation.
