# Supplementary material for: Enhancing Commercial Antibiotics with Trans-Cinnamaldehyde in Gram-Positive and Gram-Negative Bacteria: An In Vitro Approach
Source: Plants (Basel). 2024 Jan 10;13(2):192. doi: 10.3390/plants13020192 (PMC10820649; doi:10.3390/plants13020192)
Supplement: Supplementary file 1 [file plants-13-00192-s001.zip › plants-2726941-supplementary.pdf]

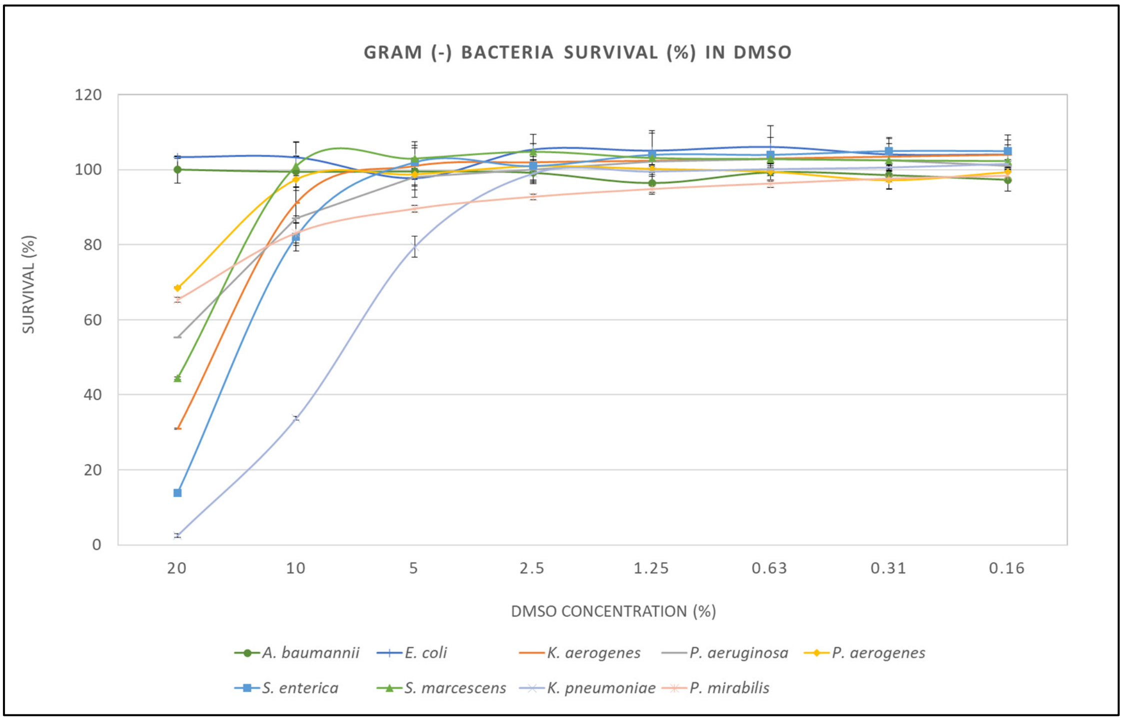

(a)

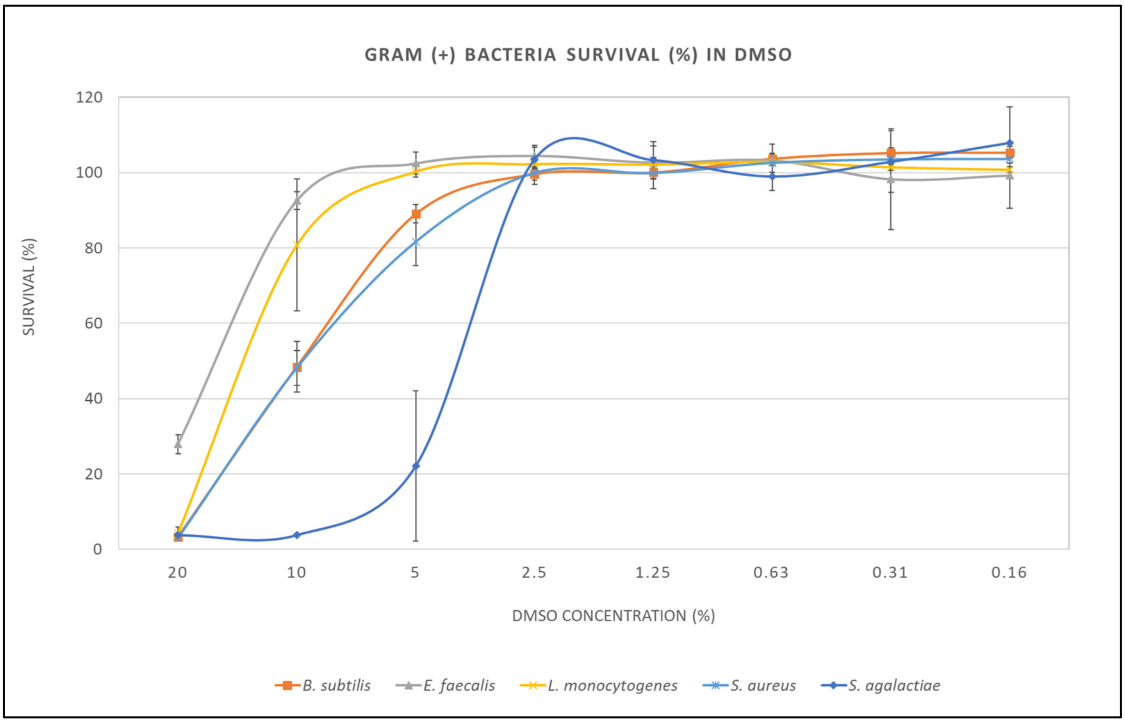

(b)

Figure S1. Survival (%) of **a)** Gram (-) bacteria and **b)** Gram (+) bacteria (B) tested in this work when exposed to different dilutions of DMSO.

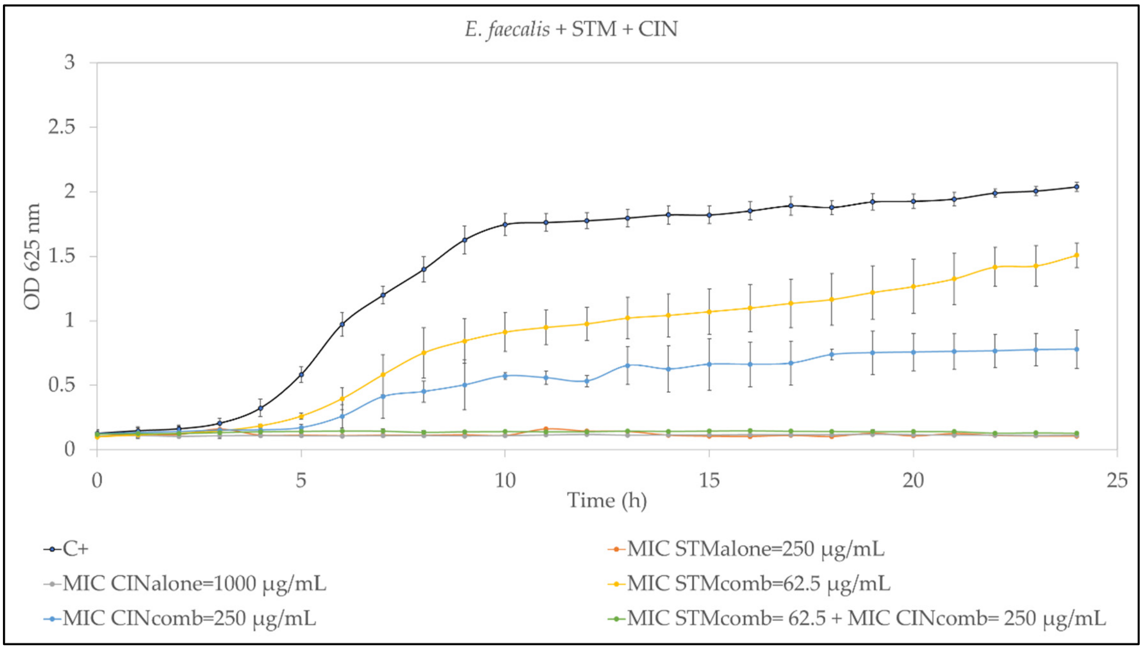

(a)

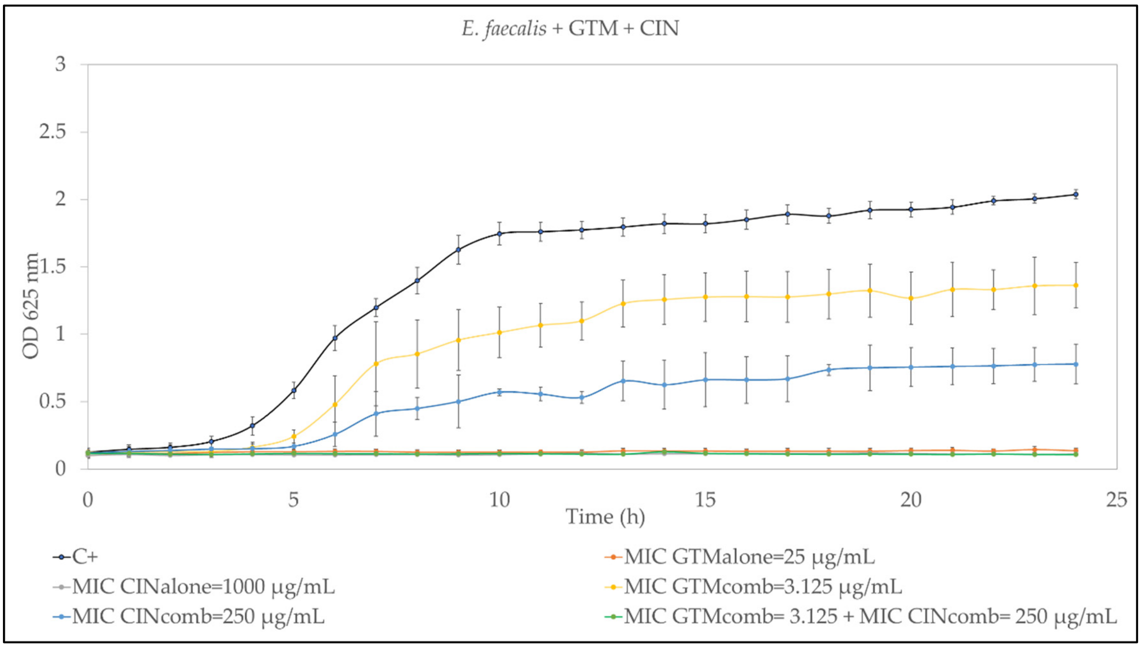

(b)

Figure S2. Kinetic study for cinnamaldehyde (CIN) and **a)** streptomycine (STM) or **b)** gentamicin (GTM) on *E. faecalis* (OD at 625 nm vs time (h)) . C+: curve for positive control. MIC CIN_{alone} and MIC ABX_{alone} are the curves for CIN and the specific ABX, respectively, when each of them was tested alone at their respective MIC. MIC ABX_{comb} is the curve for the specific ABX tested alone but added at its MIC when this and CIN were tested simultaneously. MIC CIN_{comb} is the curve for CIN tested alone but added at its MIC when this and the specific ABX were tested simultaneously. (MIC ABX_{comb}+MIC CIN_{comb}) is the cruve for the combination of the mixture of the specific ABX and CIN when tested simultaneously at their respective MICs in combination. Data are given as mean ± standard deviation.

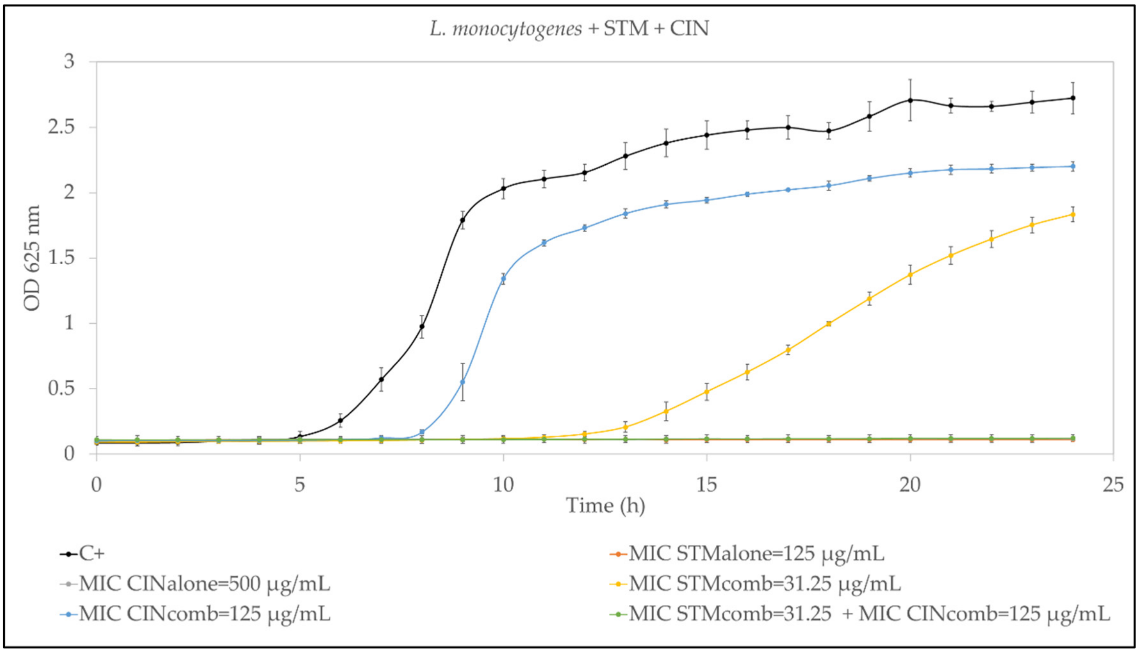

(a)

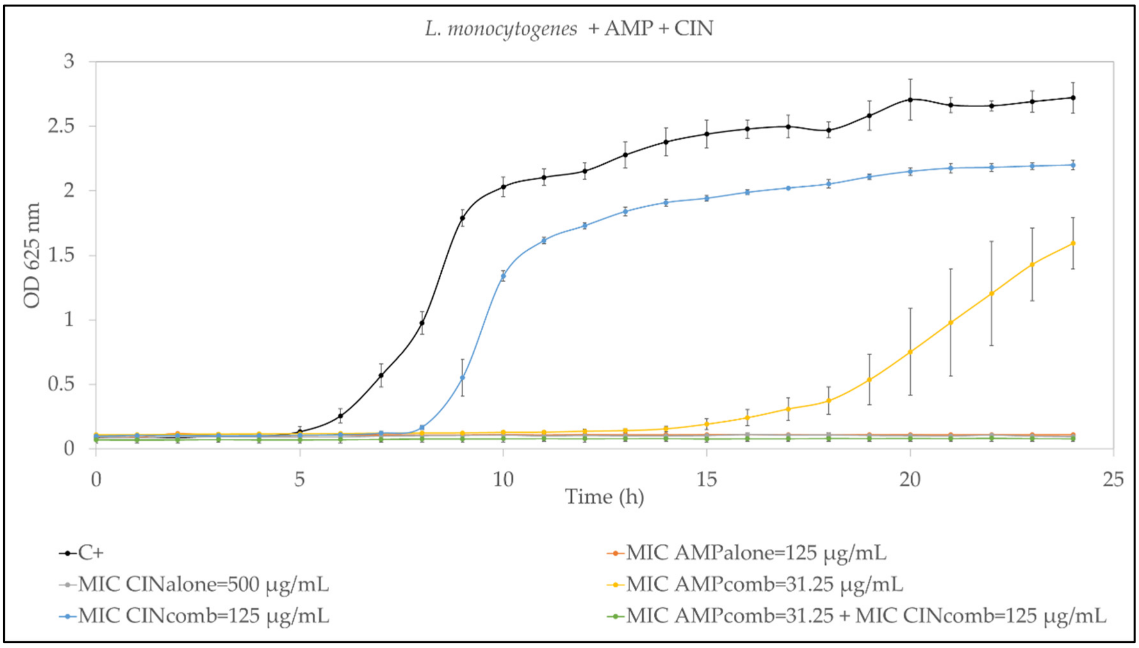

(b)

Figure S3. Kinetic study for cinnamaldehyde (CIN) and **a)** streptomycine (STM) or **b)** ampicillin (AMP) on *L. monocytogenes* (OD at 625 nm vs time (h)) . C+: curve for positive control. MIC CIN_{alone} and MIC ABX_{alone} are the curves for CIN and the specific ABX, respectively, when each of them was tested alone at their respective MIC. MIC ABX_{comb} is the curve for the specific ABX tested alone but added at its MIC when this and CIN were tested simultaneously. MIC CIN_{comb} is the curve for CIN tested alone but added at its MIC when this and the specific ABX were tested simultaneously. (MIC ABX_{comb}+MIC CIN_{comb}) is the cruve for the combination of the mixture of the specific ABX and CIN when tested simultaneously at their respective MICs in combination. Data are given as mean ± standard deviation.

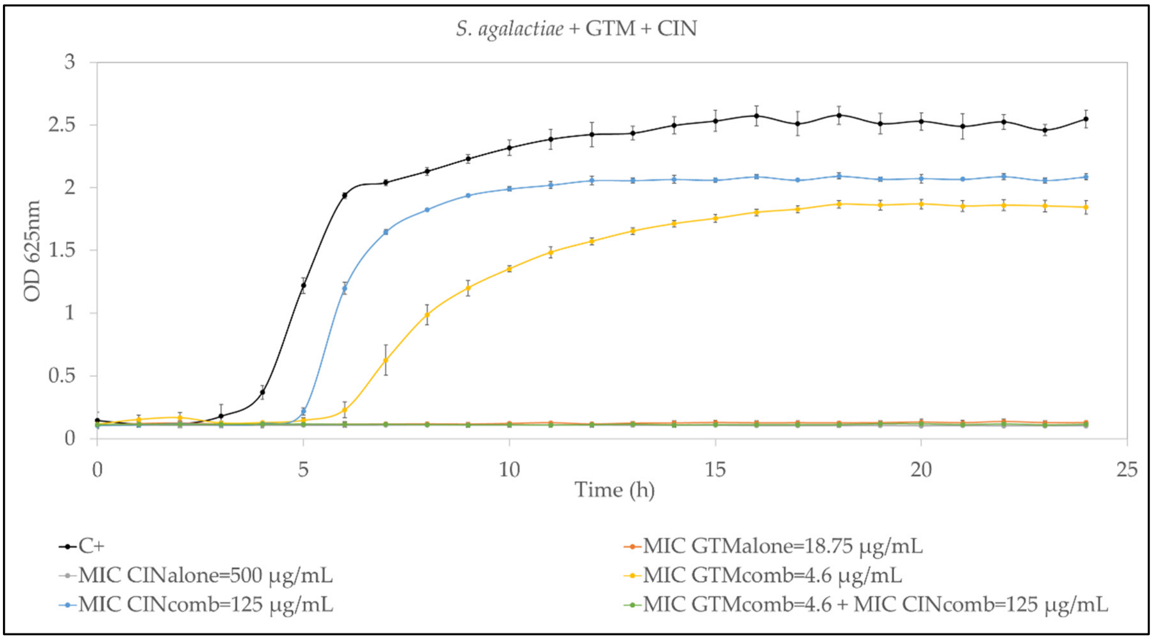

S. agalactiae + GTM + CIN
OD 625nm
Time (h)
0
0.5
1
1.5
2
2.5
3
0
5
10
15
20
25
C+
MIC CINalone=500 µg/mL
MIC CINcomb=125 µg/mL
MIC GTMalone=18.75 µg/mL
MIC GTMcomb=4.6 µg/mL
MIC GTMcomb=4.6 + MIC CINcomb=125 µg/mL

(a)

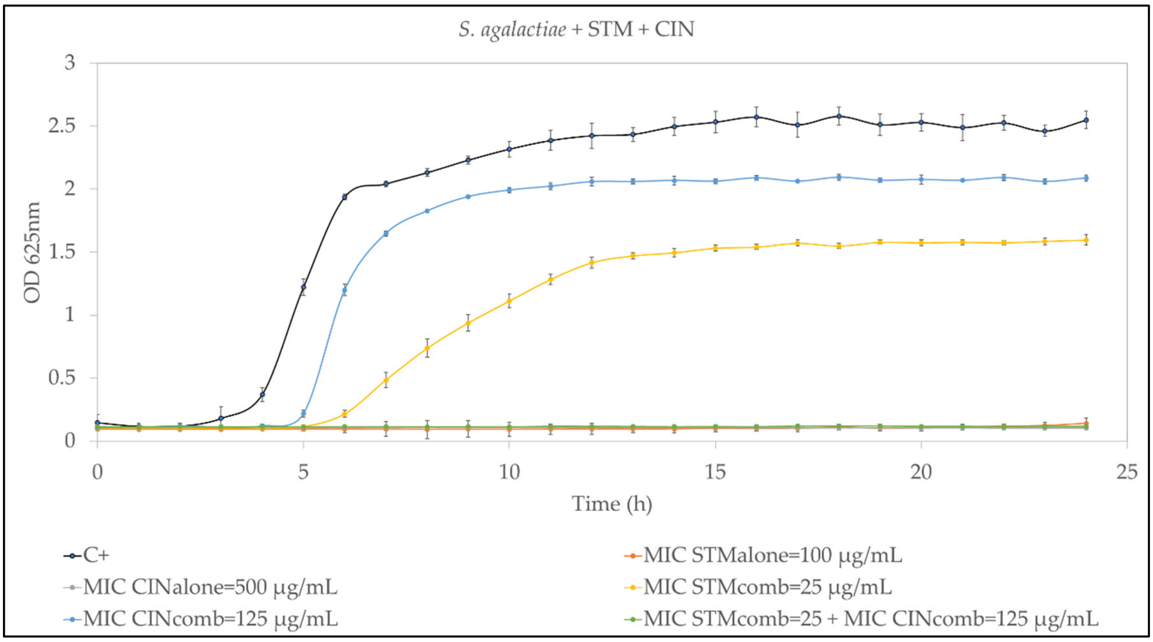

S. agalactiae + STM + CIN
OD 625nm
Time (h)
0
0.5
1
1.5
2
2.5
3
0
5
10
15
20
25
C+
MIC CINalone=500 µg/mL
MIC CINcomb=125 µg/mL
MIC STMalone=100 µg/mL
MIC STMcomb=25 µg/mL
MIC STMcomb=25 + MIC CINcomb=125 µg/mL

(b)

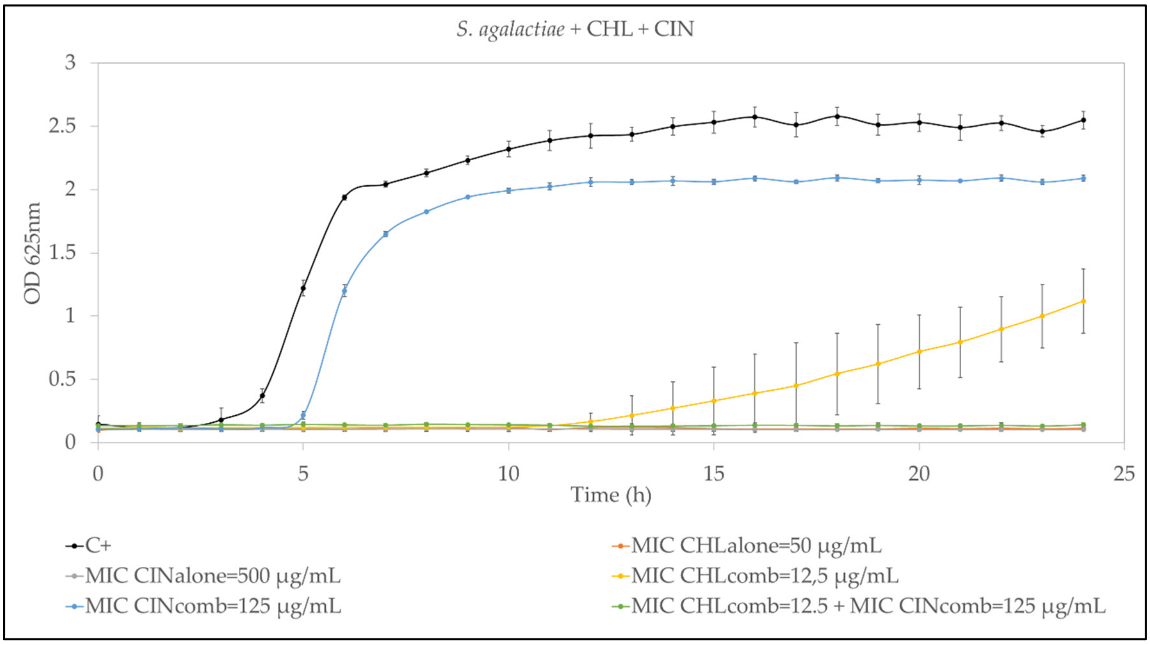

(c)

Figure S4. Kinetic study for cinnamaldehyde (CIN) and **a)** gentamicin (GTM) or **b)** streptomycin (STP) or **c)** chloramphenicol (CHL) on *S. agalactiae* (OD at 625 nm vs time (h)) . C+: curve for positive control. MIC CIN_{alone} and MIC ABX_{alone} are the curves for CIN and the specific ABX, respectively, when each of them was tested alone at their respective MIC. MIC ABX_{comb} is the curve for the specific ABX tested alone but added at its MIC when this and CIN were tested simultaneously. MIC CIN_{comb} is the curve for CIN tested alone but added at its MIC when this and the specific ABX were tested simultaneously. (MIC ABX_{comb}+MIC CIN_{comb}) is the cruve for the combination of the mixture of the specific ABX and CIN when tested simultaneously at their respective MICs in combination. Data are given as mean ± standard deviation.

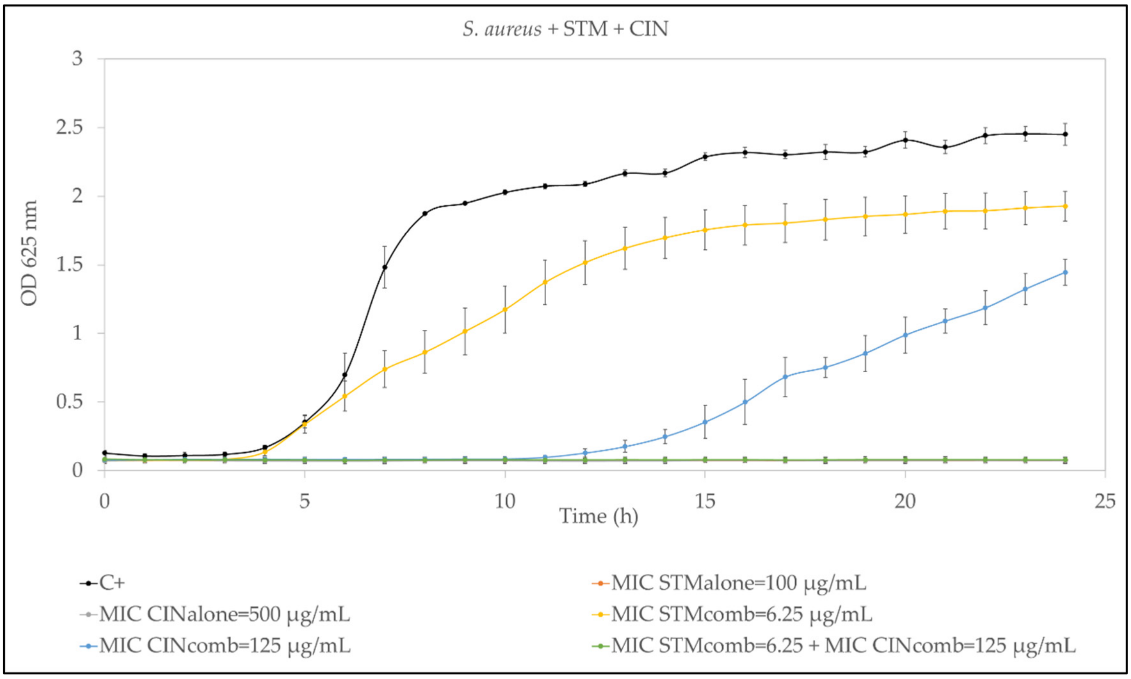

(a)

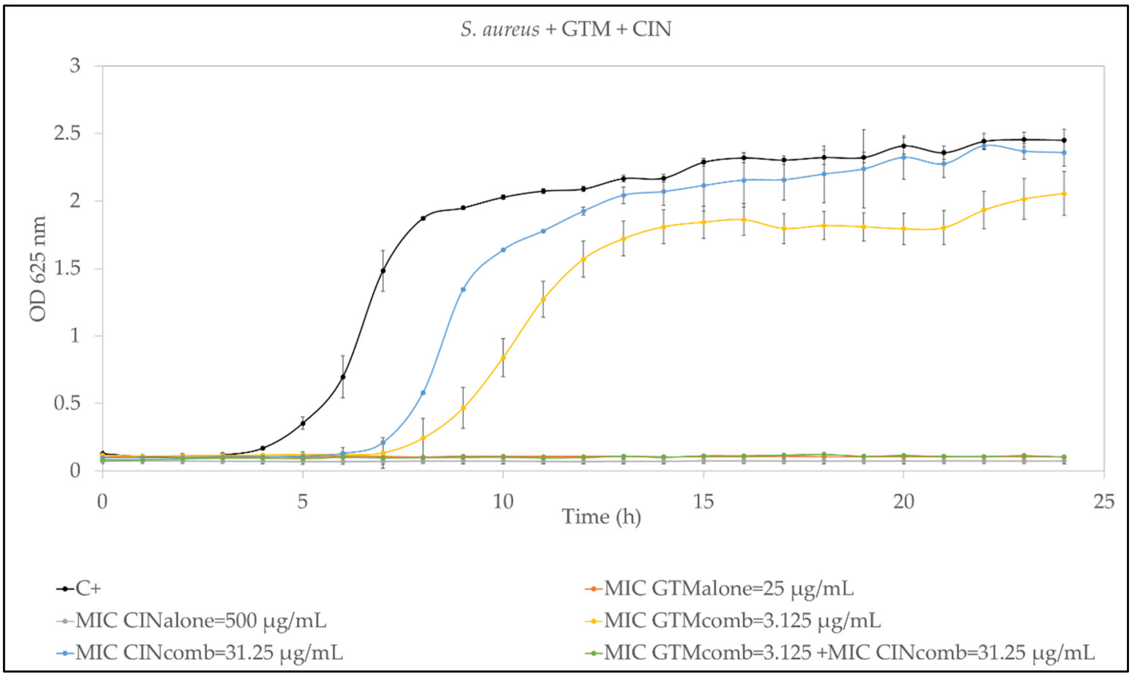

(b)

Figure S5. Kinetic study for cinnamaldehyde (CIN) and **a)** streptomycine (STM) or **b)** gentamicin (GTM) on *S. aureus* (OD at 625 nm vs time (h)) . C+: curve for positive control. MIC CIN_{alone} and MIC ABX_{alone} are the curves for CIN and the specific ABX, respectively, when each of them was tested alone at their respective MIC. MIC ABX_{comb} is the curve for the specific ABX tested alone but added at its MIC when this and CIN were tested simultaneously. MIC CIN_{comb} is the curve for CIN tested alone but added at its MIC when this and the specific ABX were tested simultaneously. (MIC ABX_{comb}+MIC CIN_{comb}) is the cruve for the combination of the mixture of the specific ABX and CIN when tested simultaneously at their respective MICs in combination. Data are given as mean ± standard deviation.

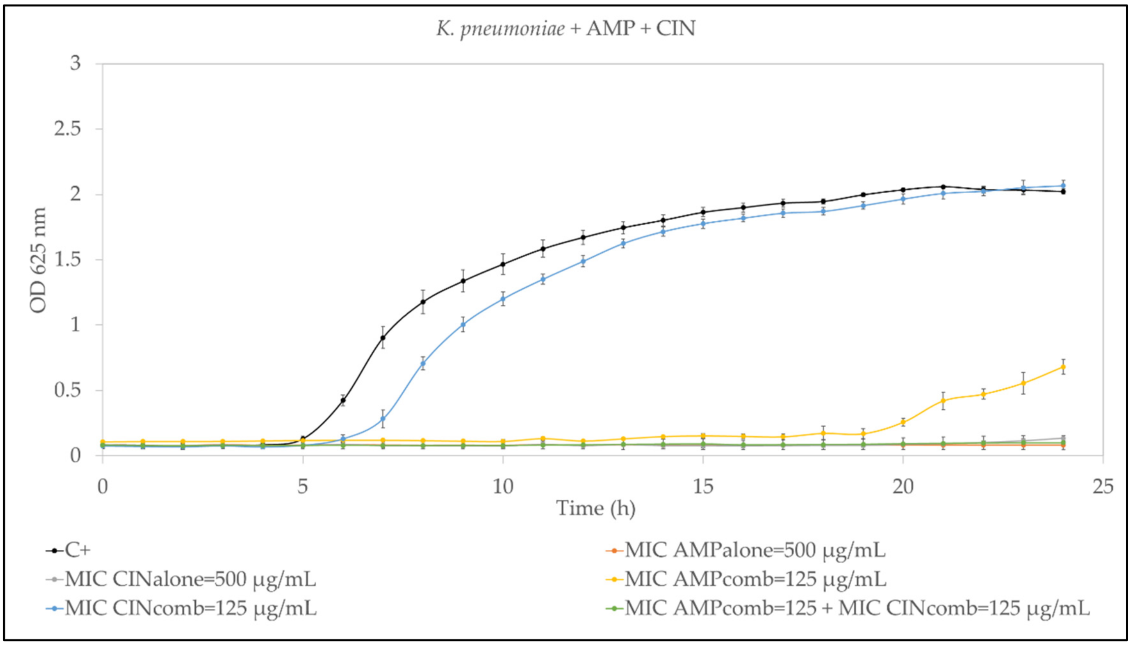

Figure S6. Kinetic study for cinnamaldehyde (CIN) and ampicillin (AMP) on *K. pneumoniae* (OD at 625 nm vs time (h)) . C+: curve for positive control. MIC CIN_{alone} and MIC ABX_{alone} are the curves for CIN and the specific ABX, respectively, when each of them was tested alone at their respective MIC. MIC ABX_{comb} is the curve for the specific ABX tested alone but added at its MIC when this and CIN were tested simultaneously. MIC CIN_{comb} is the curve for CIN tested alone but added at its MIC when this and the specific ABX were tested simultaneously. (MIC ABX_{comb}+MIC CIN_{comb}) is the cruve for the combination of the mixture of the specific ABX and CIN when tested simultaneously at their respective MICs in combination. Data are given as mean ± standard deviation.

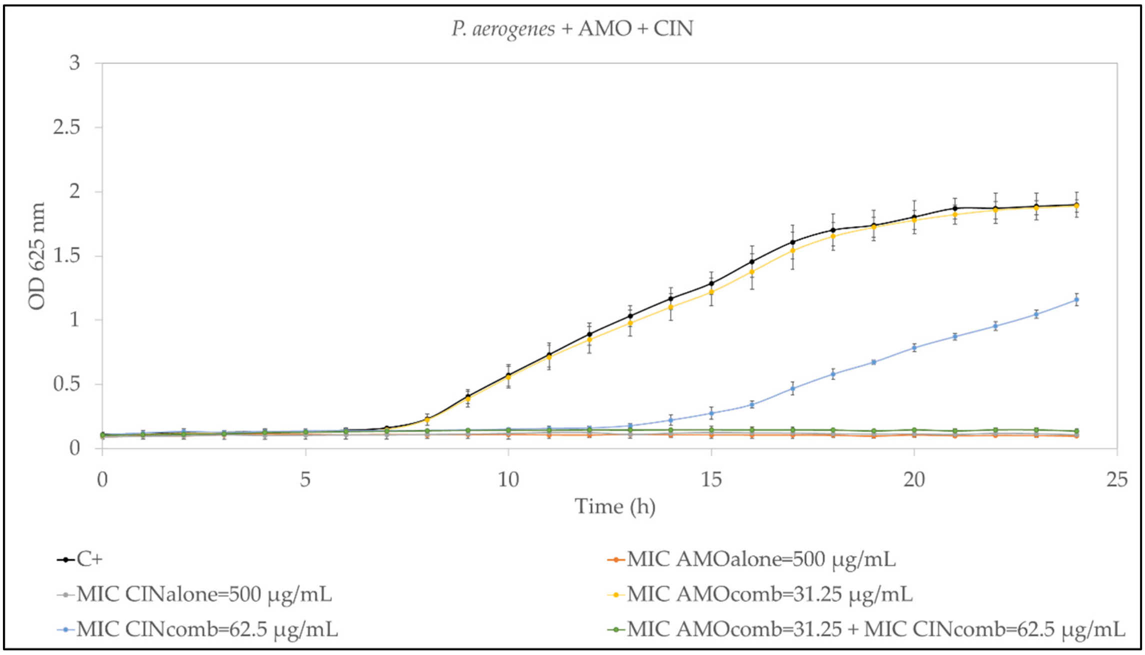

(a)

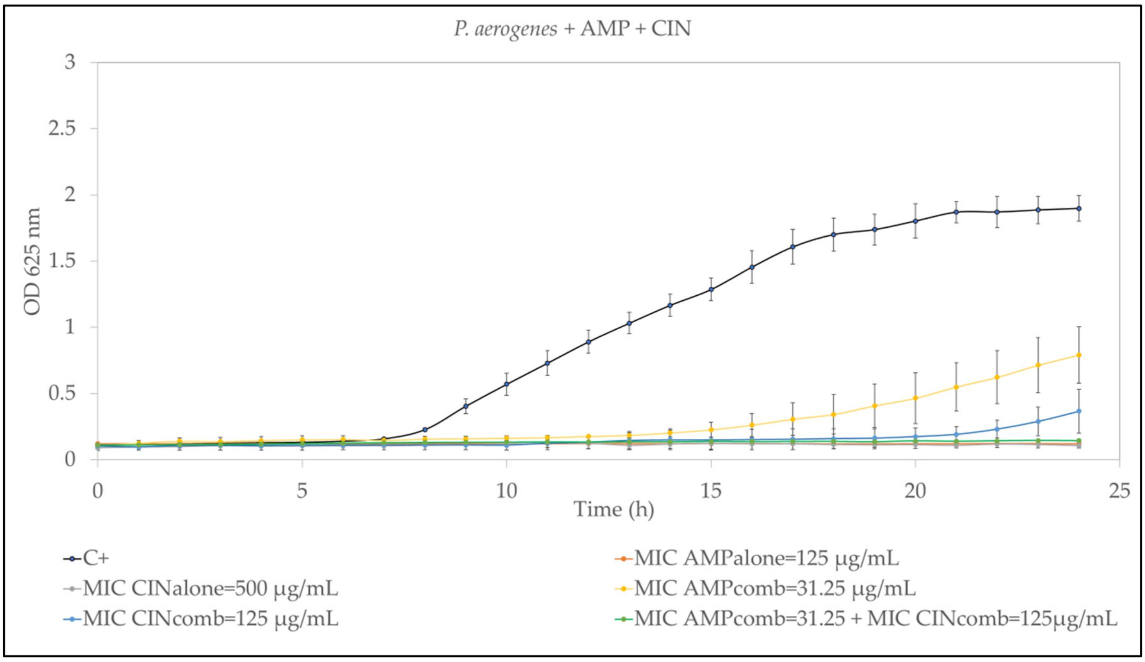

(b)

Figure S7. Kinetic study for cinnamaldehyde (CIN) and **a)** amoxicillin (AMO) or **b)** ampicillin (AMP) on *P. aerogenes* (OD at 625 nm vs time (h)) . C+: curve for positive control. MIC CIN_{alone} and MIC ABX_{alone} are the curves for CIN and the specific ABX, respectively, when each of them was tested alone at their respective MIC. MIC ABX_{comb} is the curve for the specific ABX tested alone but added at its MIC when this and CIN were tested simultaneously. MIC CIN_{comb} is the curve for CIN tested alone but added at its MIC when this and the specific ABX were tested simultaneously. (MIC ABX_{comb}+MIC CIN_{comb}) is the cruve for the combination of the mixture of the specific ABX and CIN when tested simultaneously at their respective MICs in combination. Data are given as mean ± standard deviation.

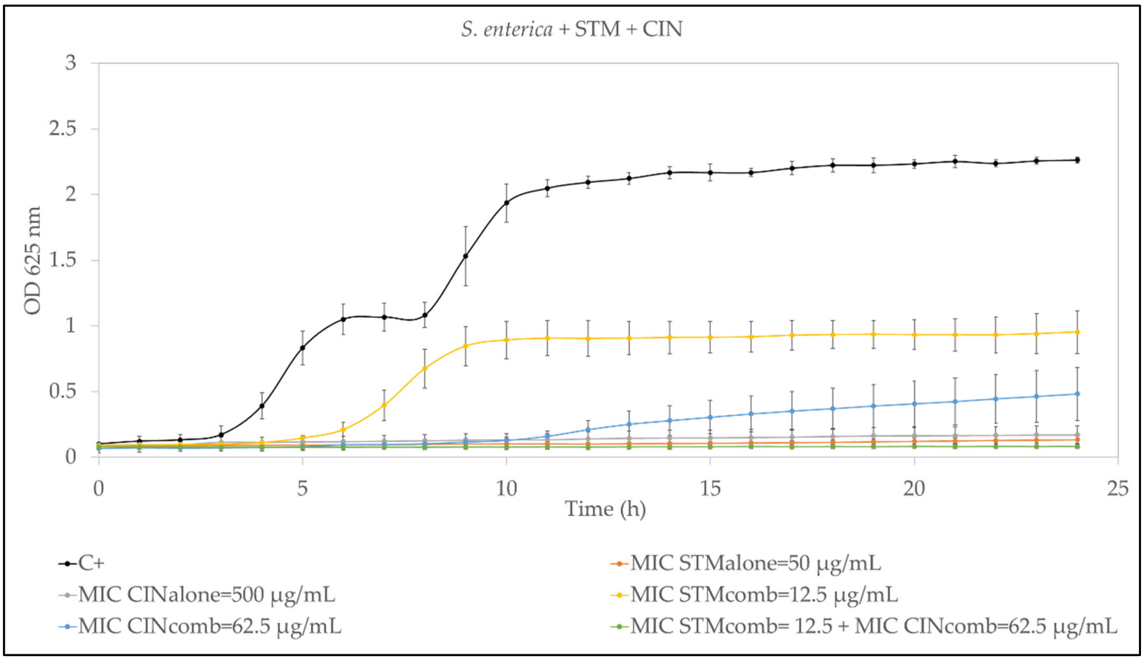

Figure S8. Kinetic study for cinnamaldehyde (CIN) and streptomycine (STM) on *S. enterica* (OD at 625 nm vs time (h)) . C+: curve for positive control. MIC CIN_{alone} and MIC ABX_{alone} are the curves for CIN and the specific ABX, respectively, when each of them was tested alone at their respective MIC. MIC ABX_{comb} is the curve for the specific ABX tested alone but added at its MIC when this and CIN were tested simultaneously. MIC CIN_{comb} is the curve for CIN tested alone but added at its MIC when this and the specific ABX were tested simultaneously. (MIC ABX_{comb}+MIC CIN_{comb}) is the cruve for the combination of the mixture of the specific ABX and CIN when tested simultaneously at their respective MICs in combination. Data are given as mean ± standard deviation.

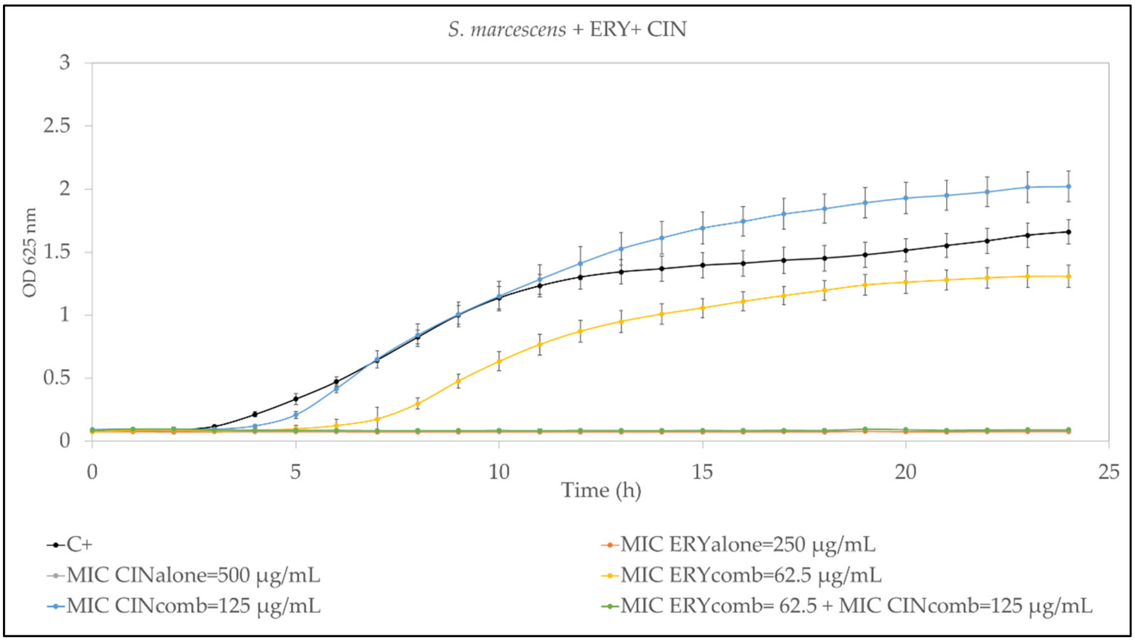

(a)

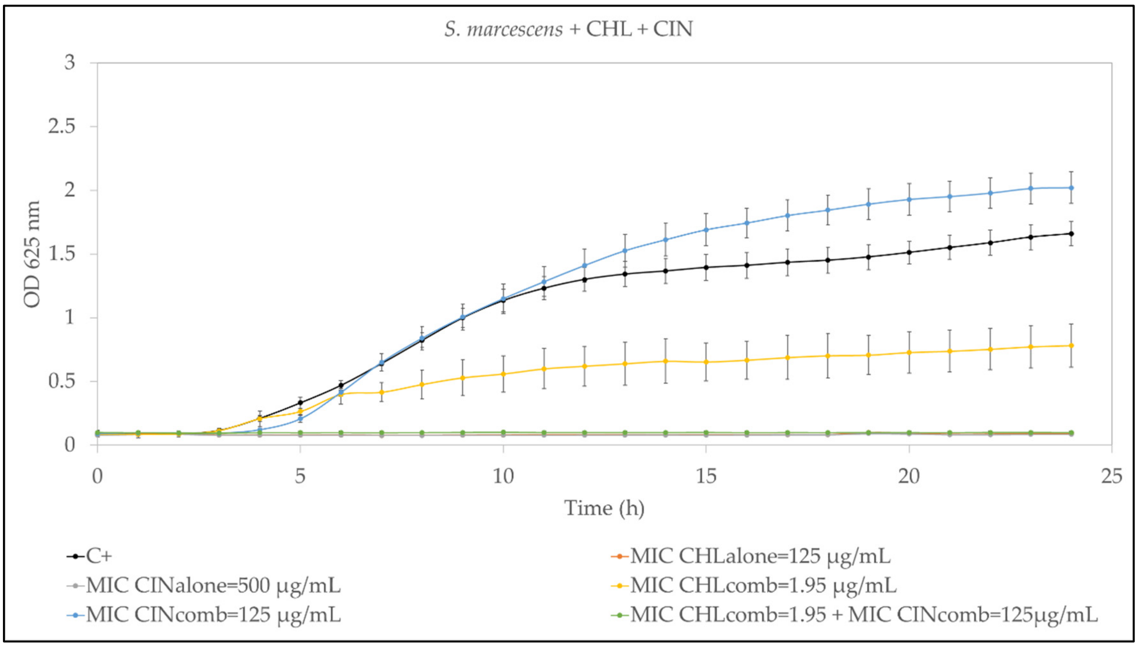

(b)

Figure S9. Kinetic study for cinnamaldehyde (CIN) and **a)** erythromycin (ERY) or **b)** chloramphenicol (CHL) on *S. marcescens* (OD at 625 nm vs time (h)) . C+: curve for positive control. MIC CIN_{alone} and MIC ABX_{alone} are the curves for CIN and the specific ABX, respectively, when each of them was tested alone at their respective MIC. MIC ABX_{comb} is the curve for the specific ABX tested alone but added at its MIC when this and CIN were tested simultaneously. MIC CIN_{comb}, is the curve for CIN tested alone but added at its MIC when this and the specific ABX were tested simultaneously. (MIC ABX_{comb}+MIC CIN_{comb}) is the cruve for the combination of the mixture of the specific ABX and CIN when tested simultaneously at their respective MICs in combination. Data are given as mean ± standard deviation.
